# Effectiveness of the BNT162b2 vaccine in preventing morbidity and mortality associated with COVID-19 in children aged 5 to 11 years: A systematic review and meta-analysis

**DOI:** 10.1371/journal.pgph.0002676

**Published:** 2023-12-04

**Authors:** Sumayyah Ebrahim, Ntombifuthi Blose, Natasha Gloeck, Ameer Hohlfeld, Yusentha Balakrishna, Rudzani Muloiwa, Andy Gray, Andy Parrish, Karen Cohen, Ruth Lancaster, Tamara Kredo

**Affiliations:** 1 Department of Surgery, Nelson R. Mandela School of Medicine, College of Health Sciences, University of KwaZulu-Natal, Durban, South Africa; 2 Cochrane South Africa, South African Medical Research Council, Cape Town, South Africa; 3 Biostatistics Research Unit, South African Medical Research Council, Durban, South Africa; 4 Department of Paediatrics and Child Health, University of Cape Town, Cape Town, South Africa; 5 Division of Pharmacology, Discipline of Pharmaceutical Sciences, University of KwaZulu-Natal, Durban, South Africa; 6 National Essential Medicines List Ministerial Advisory Committee on COVID-19 Therapeutics, National Department of Health, Pretoria, South Africa; 7 Department of Internal Medicine, Walter Sisulu University, Mthatha, South Africa; 8 Division of Clinical Pharmacology, Department of Medicine, University of Cape Town, Cape Town, South Africa; 9 Affordable Medicines Directorate, National Department of Health, Pretoria, South Africa; 10 Division of Clinical Pharmacology, Department of Medicine, and Division of Biostatistics and Epidemiology, Department of Global Health, Faculty of Medicine and Health Sciences, Stellenbosch University, Cape Town, South Africa; 11 Health Systems Research Unit, South African Medical Research Council, Cape Town, South Africa; PLOS: Public Library of Science, UNITED STATES

## Abstract

A rapid systematic review, based on Cochrane rapid review methodology was conducted to assess the effectiveness of two 10μg doses of BNT162b2 vaccine in preventing morbidity and mortality associated with COVID-19 in children aged 5 to 11 years. We searched the Cochrane Library COVID-19 study register, the COVID-NMA living review database and the McMaster University Living Evidence Synthesis for pre-appraised trials and observational studies up to 7 December 2022. Records were screened independently in duplicate. Where appraisal was not available, these were done in duplicate. Meta-analysis was conducted using RevMan 5.3 presenting risk ratios/odds ratios/inverse vaccine efficacy with 95% confidence intervals (CI). GRADE for assessing the overall certainty of the evidence was done in Gradepro. We screened 403 records and assessed 52 full-text articles for eligibility. One randomised controlled trial (RCT) and 24 observational studies were included. The RCT reported that BNT162b2 was likely safe and 91% efficacious, RR 0.09 (95% CI 0.03 to 0.32) against incident COVID-19 infection (moderate certainty evidence). In absolute terms, this is 19 fewer cases per 1,000 vaccines delivered (ranging from 15 to 21 fewer cases). Observational studies reported vaccine effectiveness (VE) against incident COVID-19 infection of 65% (OR 0.35, 95% CI 0.26 to 0.47) and 76% against hospitalisation (OR 0.24, 95% CI 0.13 to 0.42) (moderate certainty evidence). The absolute effect is 167 fewer cases per 1,000 vaccines given (ranging from 130 fewer to 196 fewer cases) and 4 fewer hospitalisations per 10,000 children (from 3 fewer to 5 fewer hospitalisations). Adverse events following vaccination with BNT162b2 were mild or moderate and transient. The evidence demonstrated a reduction in incident COVID-19 cases and small absolute reduction in hospitalisation if a two-dose BNT162b2 vaccine regimen is offered to children aged 5 to 11 years, compared to placebo. **PROSPERO registration:** CRD42021286710.

## Introduction

On 10 September 2021, the South African Health Products Regulatory Authority (SAHPRA) approved the use of the BNT162b2 (Pfizer–BioNTech; tozinameran) COVID-19 vaccine for children aged 12 years and older [1]. In that week, 14.7% of new COVID-19 cases reported were in the 12 to 17-year age group, and from mid-October 2021, 9.2% of the total number of COVID-19 cases reported since the beginning of the pandemic were reported in children and adolescents aged 10 to 19 years. The goal of vaccination of children and adolescents against COVID-19 is to shorten the duration of symptoms, limit transmission, allow an earlier return to school and reduce severe disease, while minimising health system overload [2, 3]. Adolescents with underlying conditions such as diabetes, cancer, human immunodeficiency virus (HIV) infection and obesity are at greater risk of death from COVID-19 than those without these conditions [2], and COVID-19 vaccination may be particularly important for these higher-risk adolescents.

Data from the South African National Institute for Communicable Diseases (NICD) in children aged 18 years and younger admitted with COVID-19 showed that compared to children with no comorbidities; the risk of mortality was increased in children with one or more comorbidities (adjusted odds ratio [aOR] 12.09, 95% confidence interval [CI] 4.19 to 34.89). These comorbidities were asthma/chronic pulmonary disease, hypertension, diabetes mellitus, HIV and previous tuberculosis (TB) [4]. A systematic review exploring poor prognostic factors for COVID-19 in children and adolescent found that congenital heart disease, chronic pulmonary disease, neurological diseases, and obesity were associated with unfavourable outcomes [5].

In South Africa (SA), the primary schedule offered for children and adolescents (12 to 17 years) is the BNT162b2 vaccine, whereas adults (18 years and older) can choose either the two-dose BNT162b2 or two doses of COVID-19 Vaccine Janssen. From 9 December 2021, all children 12 years and older were eligible to receive two doses of the BNT162b2 vaccine, administered 42 days apart [6]. As of 27 February 2023, children aged 5 to 11 years at risk of developing severe COVID-19 will be eligible to receive two-dose BNT162b2 in South Africa [7]. The BNT162b2 vaccine is a lipid nanoparticle formulation containing nucleoside-modified mRNA encoding the severe acute respiratory syndrome coronavirus 2 (SARS-CoV-2) viral spike glycoprotein [8]. In large, multinational, randomised controlled trials, BNT162b2 had an acceptable safety profile characterised by transient mild-to-moderate injection-site pain, fatigue, and headache. It was more reportedly immunogenic among 12 to 15-year-olds than among young adults (16 to 25-year olds). Vaccine efficacy was reported to be 95 to 100% in preventing COVID-19 from seven days to about two months after the second dose [9, 10].

The World Health Organization (WHO) recommends that countries consider using BNT162b2 in children aged 5 to 17 years only when high vaccine coverage (primary series and boosters) has been achieved in the higher priority-use groups. Countries should consider the individual and population benefits of immunising children and adolescents in their specific epidemiological and social context [11]. The Centers for Disease Control and Prevention (CDC) recommends the BNT162b2 vaccine for persons 5 to 11 years of age in the United States (US) under the US Food and Drug Administration’s (FDA) Emergency Use Authorization [12].

This review aimed to assess the effectiveness of the BNT162b2 vaccine in preventing morbidity and mortality associated with COVID-19 in children aged 5 to 11 years old. Our findings informed evidence-based recommendations by the South African National Department of Health.

## Methods

We used a pre-specified protocol following the Cochrane guidelines for rapid reviews [13] (**PROSPERO registration:** CRD42021286710). The eligibility criteria for the review were developed *a priori* and comprised the following components:

- **Population:** Children 5 to 11 years of age (sub-group population: children 5 to 11 years of age with comorbidities).
- **Intervention**: BNT162b2 (Pfizer-BioNTech) COVID-19 vaccine, two-doses, 10μg, intramuscular (IM).
- **Comparators**: Placebo or no vaccine.
- **Outcomes**:
- Symptomatic laboratory confirmed COVID-19,
- Hospitalisation due to COVID-19,
- COVID-19 related death,
- Serious adverse events, other (non-serious) adverse events.

Randomised controlled trials (RCTs) and observational studies were sought. We searched the McMaster University Living Evidence Synthesis (https://www.mcmasterforum.org/) on 23 June 2022 and 29 November 2022, the Cochrane Library COVID-19 study register (https://covid-19.cochrane.org/) on 23 June 2022 and 07 December 2022, and the COVID-NMA.com living review [14] database (www.covid-nma.com) website on 27 June 2022 for trials and observational studies. These databases regularly and systematically search PubMed, Embase, MedRxiv, WHO’s International Clinical Trial Registry Platform (ICTRP) and clinicaltrials.gov. Search terms and strategy used are found in S1 Table. Screening of records, and selection of articles was done independently by one reviewer (SE) and then cross-checked by a second reviewer (NG). Data extraction was done by two reviewers (SE) and (NG) and cross-checked by a third reviewer (NB). The main characteristics of the included studies are shown in S2 Table (study, population and intervention characteristics, outcomes and RoB assessments).

We used Review Manager (RevMan) 5 software to perform the analyses. If pooled synthesis of data was possible (i.e., when two or more studies that were clinically and methodologically similar were identified), a meta-analysis was conducted using a random-effects model. The random effects model accounts for both within-study variability (sampling error) and between-study variability (heterogeneity) when estimating the overall effect size [15]. We considered the methodological issues with pooling observational study data which presents potentially increased risk of bias and higher heterogeneity [16]. However, in the context of the available data, use of quantitative synthesis, acknowledging potential bias, is preferable to qualitative or quasi-quantitative interpretation of the results [17]. We reported risk ratios (RR) and odds ratios (OR) for dichotomous data with 95% CIs. If outcome data was reported in other ways (such as a median that was not possible to transform to a mean) or if outcome data was still missing after attempts to contact the study authors (e.g. effect measures not reported or just reported as VEs with 95% CIs) we reported these results narratively. The risk of bias (RoB 2.0) assessment for one included trial [18] was obtained from the COVID-NMA website (https://covid-nma.com/vaccines/index.php?search_by=2&search_input=walter&submit=Validate#moteur_recherche) [19], and we obtained ROBINS-I assessment for observational studies from the McMaster University Living Evidence Synthesis [20]. Where not already available, the COVID-19 vaccine adapted ROBINS-I tool was used and appraisal performed in duplicate. The Grading of Recommendations, Assessment, Development and Evaluations (GRADE) framework was used to assess the overall certainty of the evidence across each outcome, taking into consideration factors that may decrease our confidence in the study finding, including risk of bias, inconsistency, indirectness, imprecision, and publication bias [21].

## Results

### Search results

**Fig 1:**
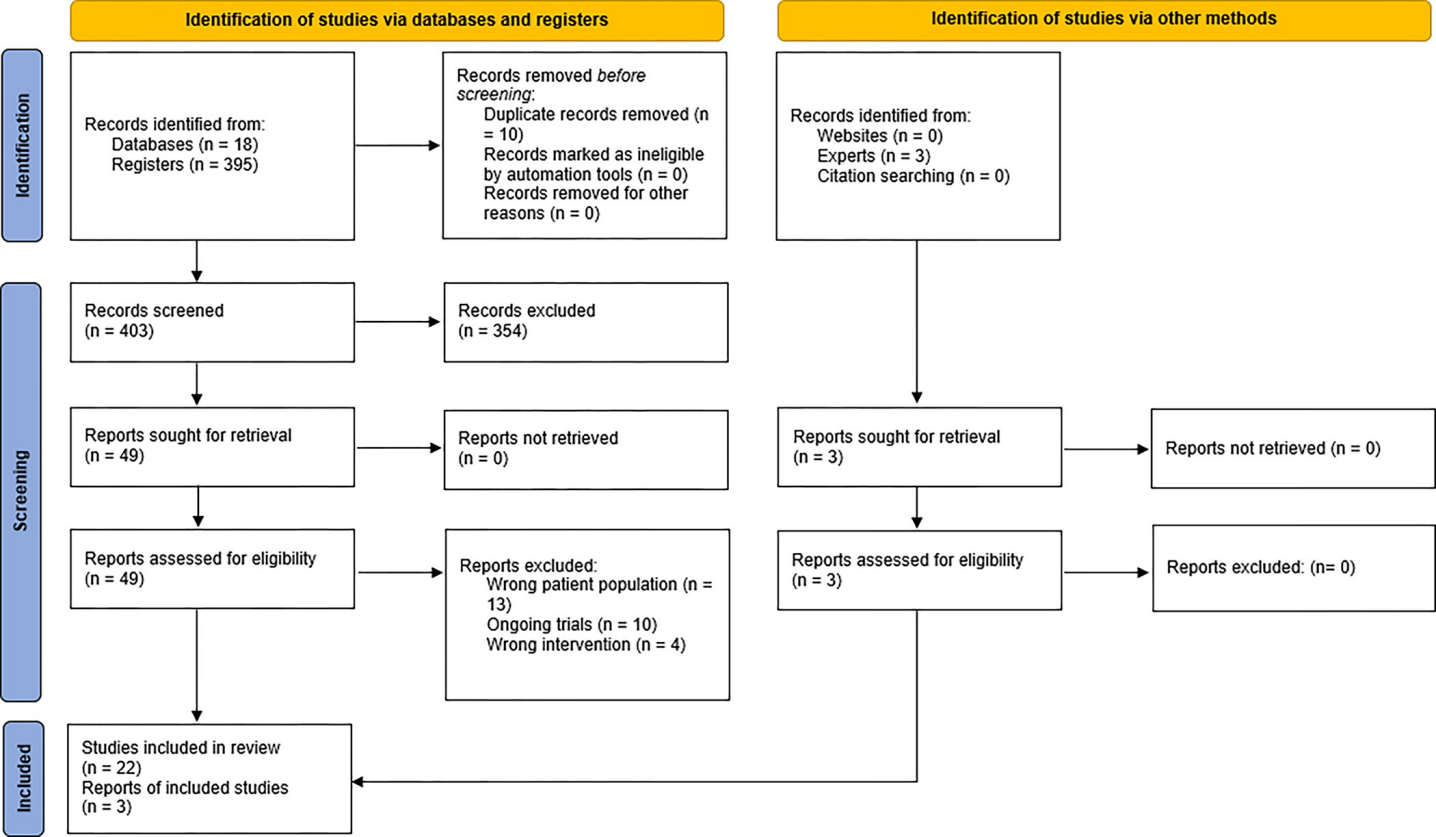
PRISMA 2020 flow diagram for review.

The updated search yielded 413 articles from three databases and three additional records were identified by the National Essential Medicines List (NEML) Ministerial Advisory Committee (MAC) on COVID-19 Therapeutics. Fifty-two studies were assessed for eligibility. One RCT and 24 observational studies were included. Seventeen studies were excluded for incorrect population or intervention [22–38], and 10 were excluded based on being ongoing trials. Fig 1 shows the PRISMA flow diagram of the search. S3 Table represents ongoing trials that will be monitored for publication as well as studies under consideration for inclusion. S4 Table lists the characteristics of excluded studies.

### Description of the studies

#### Randomised controlled trial

One trial (published) [18] conducted in the US and Europe investigated the immunogenicity, safety, and efficacy, of two doses of the BNT162b2 vaccine compared to no vaccine/placebo in children aged 5 to 11 years (n = 2,268).

#### Observational studies

There were 24 observational studies (pre-prints and published; 2021–2022) conducted in the US [39–48], Israel [49, 50], Canada [51, 52], Italy [53–57], Qatar [58], Singapore [59], South Korea [60], Japan [61] and Australia [62]. Of these, 16 investigated the safety, and effectiveness of two doses of the BNT162b2 vaccine compared to no vaccine/placebo in children aged 5 to 11 years [39–43, 45, 46, 48–53, 56, 58, 59]. The remaining eight studies were vaccine surveillance studies with no active comparator group [44, 47, 54, 55, 57, 60–62]. The follow-up duration ranged from 14 to 90 days. Studies were conducted during the Delta–Omicron dominance period in each setting. The analytic sample sizes ranged from 374 to 2 965 918 participants, and ages ranged from 6 months to 18 years. Outcomes assessed in the included studies include symptomatic laboratory confirmed COVID-19 [18, 39, 40, 42, 46, 49, 50, 52, 53, 56, 58, 59]; hospitalisation [39, 41, 43, 45, 46, 50, 51, 53, 59]; COVID-19 related death [46, 53]; serious adverse events [18, 44, 57, 60], and other adverse events [18, 44, 47, 54, 55, 57, 60–62]. Although eligible, the Simmons 2022 *et al*. study [51] was excluded from the main analysis due to lack of outcome data for the 5–11-year-old age group. The study by Zambrano 2022 *et al*. [48] evaluated vaccine effectiveness by comparing the odds of being fully vaccinated with two-doses of BNT162b2 vaccine versus being unvaccinated in Multisystem Inflammatory Syndrome in Children (MIS-C) case patients compared to hospital-based controls who tested negative for COVID-19. MIS-C is associated with severe COVID-19 infection and vaccination with two-doses of BNT162b2 may reduce the likelihood of MIS-C hospitalisation [48]. S2 Table presents a description of included studies.

### Appraisal of trials and observational studies

#### Randomised controlled trial (Cochrane ROB 2.0) [n = 1]

The Walter *et al*. [18] trial had an overall risk of bias of ‘some concerns’ due to deviations from the planned intervention. The authors’ use of the per-protocol analysis of efficacy and immunogenicity outcomes was considered inappropriate since the intention was to assess the effect of the assignment to the intervention. The reasons for exclusion of outcomes were 14 [0.9%] *vs*. 10 [1.3%] did not receive two doses; 64 [4.2%] *vs*. 11 [1.5%] had important protocol deviations as determined by the investigator or did not complete one-month follow-up after the second dose. These deviations were a concern for confirmed symptomatic COVID-19 and confirmed severe COVID-19. All other domains had a low risk of bias [19].

#### Observational studies (ROBINS-I) [n = 24]

Of the 24 observational studies, 19 studies [39, 41–45, 47, 48, 50–52, 53–55, 56–57, 59–62] had a serious risk of bias and five with moderate risk of bias [40, 46, 49, 53, 58]. Studies with a serious risk of bias were downgraded for lack of adjustment for comorbidities, exclusion of previously infected participants, lack of an objective method for ascertainment of vaccination status, and incorrect index date of medical events. Those with moderate risk of bias were downgraded for study design (cohort) as people in these studies who get vaccinated may differ in health-seeking behaviour from people who do not get vaccinated [20]. These studies were also downgraded due to the lack of an objective method to ascertain vaccination status and not accounting for the non-immune period, implying evidence of residual bias. See S1 Fig- for ROBINS-I assessments of observational studies.

### Effects of the intervention

The Summary of Findings (SoFs) in Tables 1 and 2 summarise the effects of the intervention for each of the following outcomes. We sought data from the included studies on vaccine efficacy, safety, and effectiveness in children with comorbidities, but this was not available.

**Table 1:** BNT162b2 covid-19 vaccine summary of findings table (RCT).

Outcomes	Anticipated absolute effects* (95% CI)		Relative effect (95% CI)	№ of participants (studies)	Certainty of the evidence (GRADE)
	Risk with no vaccine	Risk with BNT162b2 (Pfizer-BioNTech) COVID-19 vaccine			
**Symptomatic laboratory confirmed COVID-19**	21 per 1,000	**2 per 1,000** (1 to 7)	**RR 0.09** (0.03 to 0.32)	2261 (1 RCT)	⨁⨁⨁◯ Moderatea
**Hospitalisation—not reported**	-	-	-	-	-
**COVID-19 related death—not reported**	-	-	-	-	-
**Serious adverse events**	*Phase 1*: 16 participants received two 10-μg doses of BNT162b2 vaccine. There were no serious adverse events reported. *Phase 2–3*: Serious adverse events were reported in 0.1% of BNT162b2 recipients (2/1,518) and 0.1% of placebo recipients (1/750), from the first dose to one month after the second dose.			2268 (1 RCT)	⨁⨁◯◯ Lowb
**Adverse events (non-serious)**	92 per 1,000	**109 per 1,000** (84 to 143)	**RR 1.19** (0.91 to 1.55)	2268 (1 RCT)	⨁⨁⨁◯ Moderatec
*The risk in the intervention group (and its 95% confidence interval) is based on the assumed risk in the comparison group and the **relative effect** of the intervention (and its 95% CI). **CI:** confidence interval; **RR:** risk ratio					
**GRADE Working Group grades of evidence** **High certainty:** we are very confident that the true effect lies close to that of the estimate of the effect. **Moderate certainty:** we are moderately confident in the effect estimate: the true effect is likely to be close to the estimate of the effect, but there is a possibility that it is substantially different. **Low certainty:** our confidence in the effect estimate is limited: the true effect may be substantially different from the estimate of the effect. **Very low certainty:** we have very little confidence in the effect estimate: the true effect is likely to be substantially different from the estimate of effect.					

Explanations

Downgraded by one level for imprecision: Low number of events. Fragility index calculated at 13: the number of patients required to lose statistical significance, https://clincalc.com/Stats/FragilityIndex.aspx

Downgraded by two levels for imprecision: Low number of events

Downgraded by one level for imprecision: Wide confidence interval ranging from a 9% reduction in risk to a 55% increase in risk

**Table 2:** BNT162b2 covid-19 vaccine summary of findings table (observational).

Outcomes	Anticipated absolute effects* (95% CI)		Relative effect (95% CI)	№ of participants (studies)	Certainty of the evidence (GRADE)
	Risk with no vaccine	Risk with Pfizer-BioNTech COVID-19 vaccine (BNT162b2), 2 doses			
**Symptomatic laboratory confirmed COVID-19**	294 per 1,000	**127 per 1,000** (98 to 164)	**OR 0.35** (0.26 to 0.47)	4210407 (8 observational studies)	⨁⨁⨁◯ Moderate^a^
**Hospitalisation**	5 per 10,000	**1 per 10,000** (1 to 2)	**OR 0.24** (0.13 to 0.42)	4089598 (7 observational studies)	⨁⨁⨁◯ Moderate^b^
**COVID-19 related death**	4 per 1,000,000	**1 per 1,000,000** (0 to 7)	**OR 0.24** (0.03 to 1.93)	3673691 (2 observational studies)	⨁⨁◯◯ Low^c^
**Serious adverse events**	Kim 2022: There were no serious adverse events reported to the CVMS Hause 2021: There were 2.4% (100/4,249) serious adverse events reported to VAERS Ripabelli 2022: One severe adverse event of fever ≥39°C was reported, 0.50% (1/199)			(3 observational studies)	⨁◯◯◯ Very low^d,e,f^
**Adverse events (non-serious)**	Across all studies, the most common local adverse events were injection site pain and inflammation. Systemic adverse events commonly included fatigue and headache.			(8 observational studies)	⨁◯◯◯ Very low^f,g^

**CI:** confidence interval; **OR:** odds ratio; **RR:** risk ratio; **CVMS:** COVID-19 vaccination management system; **VAERS:** Vaccine Adverse Event Reporting System

Explanations

Downgraded by one level for serious risk of bias (ROB): Chemaitelly, Fowlkes, Lin and Sacco assessed as moderate ROB; Cohen-Stavi, Fleming-Dutra, Piché-Renaud and Tan assessed as serious ROB using ROBINS-I

Downgraded by one level for serious risk of bias (ROB): Lin and Sacco assessed as moderate ROB; Cohen-Stavi, Klein, Price, Shi and Tan assessed as serious ROB using ROBINS-I

Downgraded by two levels for imprecision: low number of events, wide confidence interval ranging from 97% reduction in risk to a 1.93-fold increase

Downgraded by two levels for serious ROB: Hause and Kim studies used passive surveillance, subject to reporting biases and/or underreporting; Ripabelli used active surveillance, however, missing information was noted for many domains using ROBINS-I

Downgraded by one level for indirectness: Hause study used passive surveillance, data on race/ethnicity not provided in >40% of VAERS reports

Downgraded by one level for imprecision: Low number of events, passive surveillance, no comparison group

Downgraded by two levels for serious ROB: Hause and Kim studies used passive surveillance, subject to reporting biases and/or underreporting; Bloise, Capponi, Malden, Ripabelli, Wood and Yoshida used active surveillance, however, missing information was noted for many domains using ROBINS-I

#### Randomised controlled trial

Walter *et al*. [18] evaluated safety and efficacy of the BNT162b2 vaccine in children aged 5 to 11 years. Hospitalisation and COVID-19 related death were not reported in the trial.

1. Symptomatic laboratory confirmed COVID-19

There were 3/1514 cases of COVID-19 infection in the two-dose BNT162b2 group (0.20%) and 16/747 in the placebo group (2.14%). A two-dose BNT162b2 vaccine regimen compared to placebo may result in small reduction in COVID-19 infection (7 days or more after the second dose), RR 0.09 (95% CI 0.03 to 0.32), n = 2,261, moderate certainty evidence. In absolute terms, this is 19 fewer cases per 1,000 vaccines delivered (ranging from 15 to 21 fewer cases). Vaccine efficacy was 91% (95% CI 68% to 97%).

1. Serious adverse events

*Phase 1*: 16 participants received two 10-μg doses of BNT162b2 vaccine. There were no serious adverse events reported.

*Phase 2–3*: There is low certainty evidence that there may be little or no difference in the occurrence of serious adverse events. Serious adverse events were reported in 0.1% of BNT162b2 recipients (2/1,518) and 0.1% of placebo recipients (1/750), from the first dose to one month after the second dose. Three serious adverse events in two participants were reported by the cut-off date (a median of 2.3 months’ follow-up); all three (post-injury abdominal pain and pancreatitis in a placebo recipient and arm fracture in a BNT162b2 recipient) were unrelated to the vaccine or placebo. No deaths or adverse events leading to withdrawal were reported.

1. Adverse events (non-serious)

*Phase 1*: Most reported local reactions were mild to moderate, and all were transient. Adverse events from the first dose through one month after the second dose were reported by 43.8% (7/16) of participants who received two 10-μg doses of BNT162b2.

*Phase 2*: From the first dose through one month after the second dose, adverse events were reported by 10.9% (166/1,518) of BNT162b2 recipients and 9.2% (69/750) of placebo recipients, RR 1.19 (95% CI 0.91 to 1.55), moderate certainty evidence.

There were more local reactions and systemic events reported in BNT162b2 recipients than placebo recipients. Injection-site pain was the most common local reaction (71% to 74% of BNT162b2 recipients) with fatigue and headache being the most frequently reported systemic events. Lymphadenopathy was reported in 10 BNT162b2 recipients (0.9%) and one placebo recipient (0.1%). There were no reported cases of myocarditis, pericarditis, hypersensitivity, or anaphylaxis in vaccine recipients. The reported reactions and events were generally mild to moderate, lasting one to two days. There were four BNT162b2 vaccine recipients who reported rashes (observed on the arm, torso, face, or body, with no consistent pattern); these rashes were mild and self-limiting.

#### Observational studies (n = 24)

1. Symptomatic laboratory confirmed COVID-19

**Fig 2:**
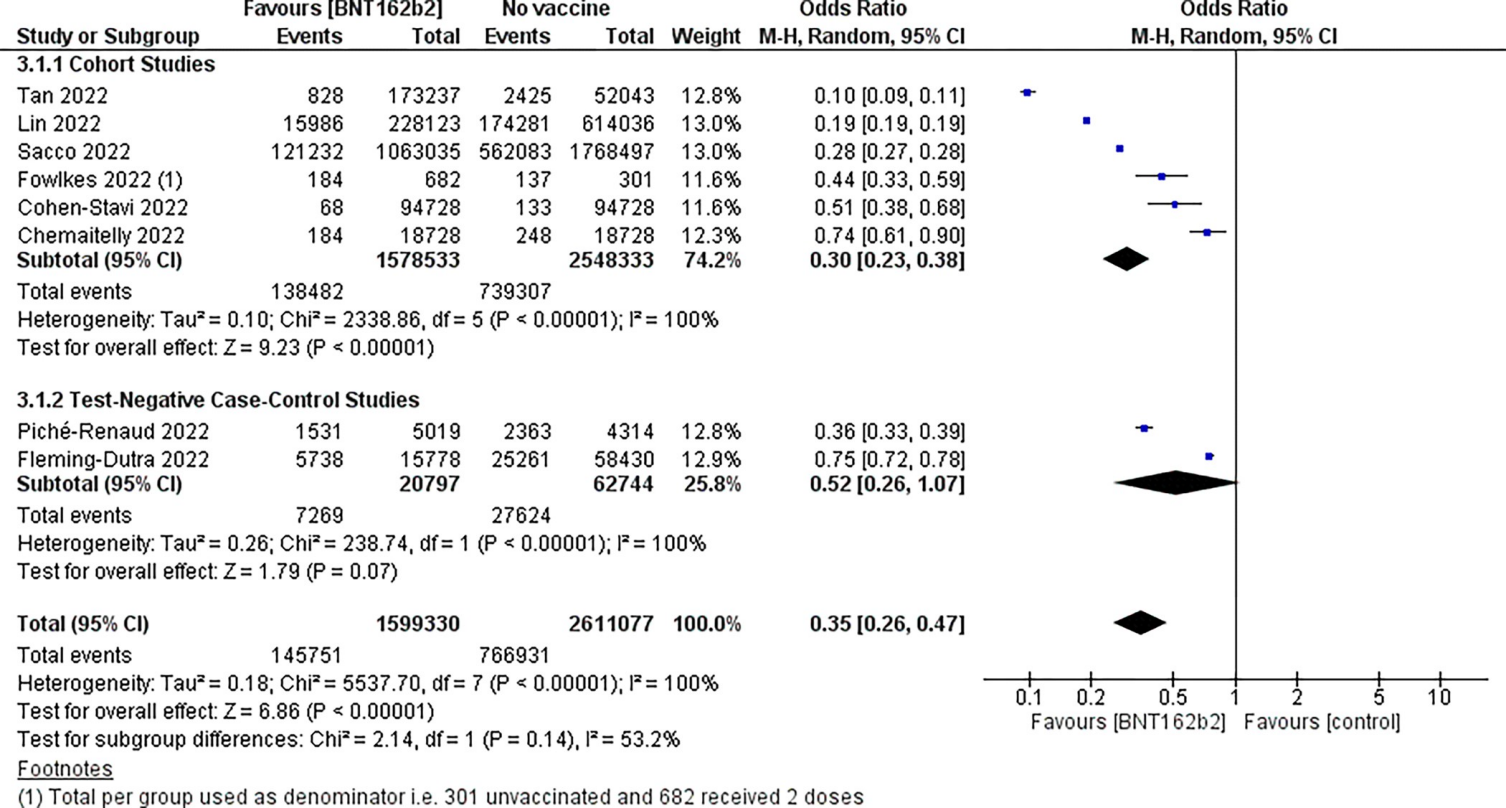
Forest plot–BNT162b2 COVID-19 vaccine *vs* no vaccine; outcome symptomatic laboratory confirmed COVID-19.

Overall, two-dose BNT162b2 vaccine compared to placebo likely results in a reduction in COVID-19 infection rates (day 14–90), OR 0.35 (95% CI 0.26 to 0.47), eight studies, n = 4,210,407, moderate certainty evidence. The absolute effect is 167 fewer cases per 1,000 vaccines given (ranging from 130 fewer to 196 fewer cases). Vaccine effectiveness was 65% (95% CI 53% to 74%). Eight studies (40, 42, 46, 50, 52, 53, 58–59) had available event and total group data to evaluate this outcome. Fig 2 shows the forest plot for this comparison. Heterogeneity was high (I^2^ = 100%) for both cohort and case-control studies, with consistency in favouring the intervention.

Three studies’ data were not included in the meta-analysis due to the methods of reporting of their data but found similar results. In the Amir *et al*., study [49] of 701,381 children aged 5–10 years; unvaccinated children aged 5 to 10 years had a 2.4-fold higher risk of infection compared to the two-dose BNT162b2 recipients (day 14 to 35 days after 2^nd^ dose), RR 2.4 (95% CI 2.2 to 2.6). In the Dorabawila *et al*. study [39] of 365,502 children aged 5 to 11 years who were fully vaccinated, the proportion of infections in vaccinated children was 4.42% (16,146/365,502). From 13 December 2021 to 30 January 2022, vaccine effectiveness (VE) against cases declined from 68% (95% CI 63% to 72%) to 12% (95% CI 6% to 16%) in this age group. Cocchio *et al*. [56] included 193,509 children aged 5 to 11 years. Overall vaccine effectiveness after two doses of BNT162b2 was 35% (95% CI 34% to 37%) and ranged from 72% (95% CI 69% to 74%) in the first week following vaccination to 23% (95% CI 20% to 26%) after ≥70 days post vaccination.

1. Hospitalisation due to COVID-19

**Fig 3:**
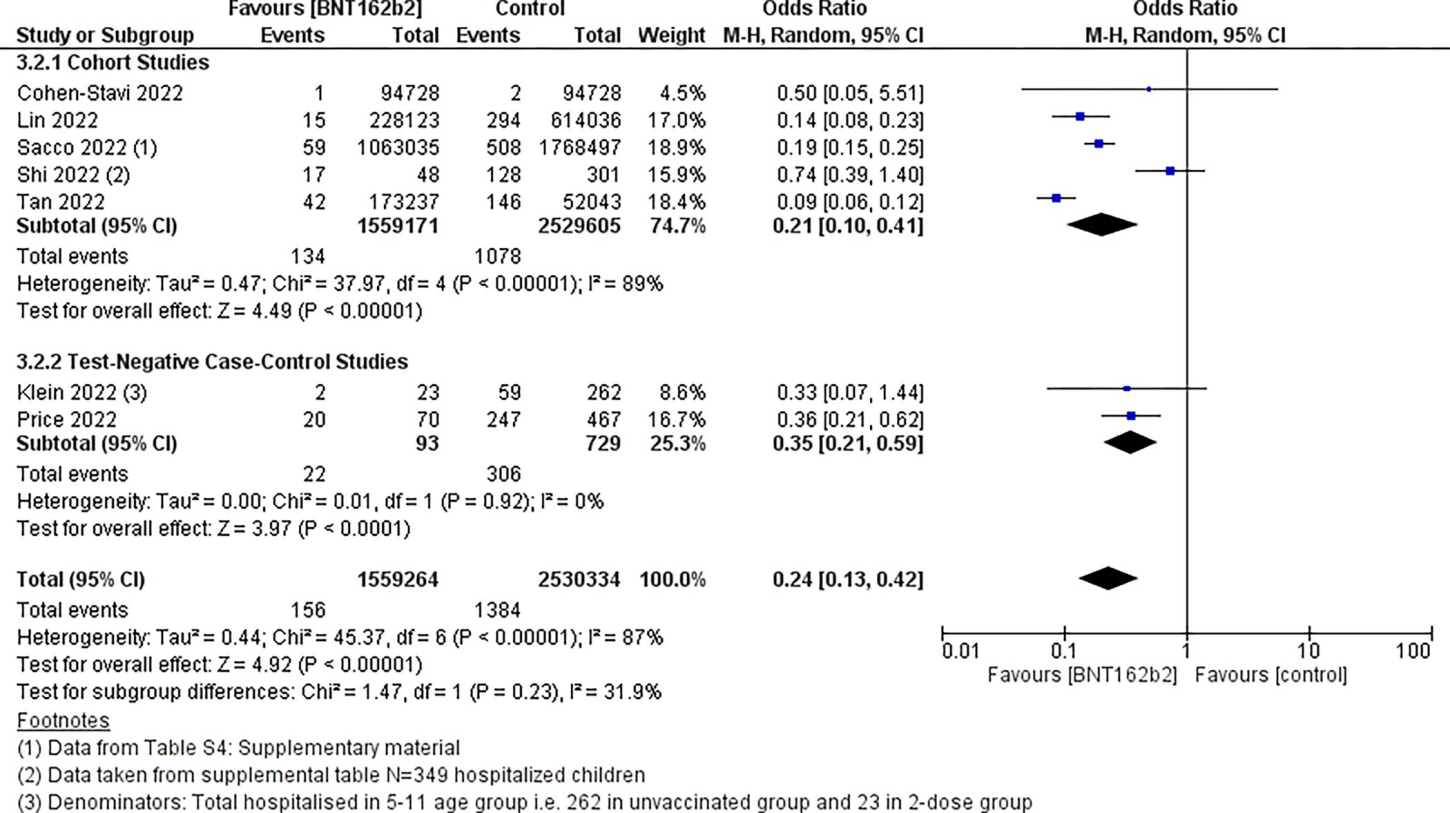
Forest plot–BNT162b2 COVID-19 vaccine *vs* no vaccine; outcome hospitalisations.

Two-dose BNT162b2 vaccine compared to placebo probably results in a small reduction in the number of hospitalisations (day 21–90) (OR 0.24, 95% CI 0.13 to 0.42), seven studies, n = 4,089,598, moderate certainty evidence. The absolute effect is four fewer hospitalisations per 10,000 children who receive a vaccination (from 3 fewer to 5 fewer hospitalisations). Vaccine effectiveness was 76% (95% CI 58% to 87%). Seven studies (41, 43, 45, 46, 50, 53, 59) had available event/total group data to evaluate this outcome. Fig 3 shows the forest plot for this comparison. Heterogeneity was high for the cohort studies (I^2^ = 89%) while there was no heterogeneity amongst the two case-control studies (I^2^ = 0%).

Two studies’ data were not included in the meta-analysis due to the methods of reporting of their data but found similar results. In a study by Dorabawila *et al*. [39] of 365,502 children aged 5 to 11 years who were fully vaccinated; from 13 December 2021 to 30 January 2022, VE against hospitalisation declined from 100% (95% CI -189% to 100%) to 48% (95% CI– 12% to 75%). In Simmons *et al*. [51] of 753 children aged 4 to 11 years who were diagnosed with COVID-19 in the study period, one vaccine dose was 32% effective against hospitalisation (aOR 0.68, 95% CI 0.28 to 1.49). In the two-dose vaccine group, less than five children were hospitalised. The study by Zambrano 2022 *et al*. [48] evaluated VE against MIS-C in hospitalised children. MIS-C is a severe hyperinflammatory condition that occurs at around 4 weeks post COVID-19 infection [48]. The study reported a VE of 78% (95% CI– 48% to 90%) against MIS-C after two-dose BNT162b2 vaccination in children aged 5 to 11 years (>28 days).

1. COVID-19 related death

Two-dose BNT162b2 vaccine compared to placebo may have little or no impact on mortality (day 84) (OR 0.24, 95% CI 0.03 to 1.93), two studies [46, 53], n = 3,673,691, low certainty evidence. There were nine deaths in the unvaccinated group (0.0004%, n = 2,382,533).

1. Serious adverse events

Hause *et al*. [44] reported on adverse events, but there was no comparator group in this study. The CDC reviewed adverse events following BNT162b2 vaccine administration reported to the Vaccine Adverse Event Reporting System (VAERS) for the period 03 November to 19 December 2021. VAERS is a passive vaccine safety surveillance system, and adverse events and health impact assessments are reported to “v-safe”, a voluntary smartphone-based safety surveillance system for adverse events after COVID-19 vaccination, in the US. There were 2.4% (100/4,249) serious adverse events reported to VAERS. These included fever, vomiting, and increased troponin levels. There were also 12 serious reports of seizure and 15 preliminary reports of myocarditis. Of these, 11 were verified (either by healthcare provider interview or medical record review) and met the case definition for myocarditis. VAERS also received two reports of death in this period; in two children, aged 5 and 6 years. Both “had complicated medical histories and were in fragile health before vaccination”. The data reviewed did not suggest a causal association between death and vaccination. Similarly, the study by Kim 2022 *et al*. [60] described adverse events as reported through the COVID-19 vaccination management system (CVMS), a web-based passive vaccine safety surveillance system and those collected via a text message-based survey sent to parents and guardians of children who had received two-doses of the BNT162b2 vaccine. For the period 5 March 2021 to 2 July 2022, CVMS received reports of 14,786 adverse events among children and adolescents aged 5 to 17 years. Following the first vaccine dose, there was one reported case of suspected myocarditis/pericarditis and one suspected case of anaphylaxis. These cases were not medically confirmed diagnoses. There were no serious adverse events reported to the CVMS following the second dose of BNT162b2 vaccination in children aged 5 to 11 years. The study by Ripabelli 2022 *et al*. [57] investigated adverse events in Italy through an active surveillance reporting system. In the period December 2021-January 2022, 199 children received at least two vaccine doses. One severe adverse event of fever ≥39°C was reported, 0.50% (1/199). This is considered very low certainty evidence.

1. Adverse events (non-serious)

Eight studies reported on adverse events [44, 47, 54, 55, 57, 60–62]. There were no comparator groups in these studies. Results are summarised in Table 3. This is also considered very low certainty evidence.

**Table 3:** Adverse events reported following BNT162b2 vaccine administration.

**STUDY ID**	**SURVEILLANCE SYSTEM USED**	**PERIOD**	**NUMBER OF CHILDREN RECEIVING TWO VACCINE DOSES AND/OR ADVERSE EVENTS REPORTED (N)**	**FINDINGS**
Bloise 2022	Telephonic survey	15 December 2021 to 11 January 2022	569 children enrolled, 449 received the second vaccine dose	• *24–48 hours after the second dose*: 51.2% reported local and/or systemic events. The most common local adverse events were injection site pain, redness, swelling and induration of the site. Systemic adverse events included fatigue, headache, fever, chills, arthromyalgia and gastrointestinal symptoms • *7 days after the second dose*: 0.8% experienced symptoms of headache, cough and lymphadenopathy • *20 days after the second dose*: No reported adverse events • Injection site reactions were more frequent after the first dose (p = 0.01), while systemic symptoms were more frequent after the second dose (p = 0.022)• Adverse events were more frequent in patients with comorbidities (p = 0.0159)
Capponi 2022	Self-administered online survey	Participants were recruited between 1 February and 28 February of 2022	579 children aged 5 to 11 years who received two vaccine doses	• 59.6% (345/579) children reported adverse events after the second vaccine dose. Pain at the injection site was the most common local adverse event, whilst fatigue, fever, chills, headache, joint and abdominal pain were the commonly reported systemic adverse events • Injection site reactions were more frequent after the first dose (p = 0.022), while systemic symptoms were more frequent after the second dose (p = 0.045)
Hause 2021	• Vaccine Adverse Event Reporting System (VAERS) • “v-safe”, a voluntary smartphone-based safety surveillance system	03 November to 19 December 2021	• VAERS: 4,429 adverse events reported• v-safe: 29,899 (with 2^nd^ dose information available)	• VAERS: Non-serious adverse events were reported in 97.6% (4,149/4,249) of the group. The most reported non-serious events were related to vaccine administration, including no adverse event (n = 1,157; 27.9%), product preparation issue (n = 925; 22.3%), and incorrect dose administered (n = 675; 16.3%). Other systemic effects included vomiting, fever, headache, syncope, dizziness, fatigue, nausea and urticaria • v-safe: After the second dose, a total of 17,180 (57.5%) local and 12,223 systemic (40.9%) reactions (including injection-site pain, fatigue, or headache) were reported
**STUDY ID**	**SURVEILLANCE SYSTEM USED**	**PERIOD**	**ADVERSE EVENTS REPORTED OR NUMBER OF CHILDREN RECEIVING TWO VACCINE DOSES (N)**	**FINDINGS**
Kim 2022	• COVID-19 vaccination management system (CVMS) • Text message-based survey	• CVMS: 05 March 2021 to 02 July 2022 • Text message-based survey: 31 March 2022 to 20 June 2022	• CVMS: 14,786 adverse events reported (5 to 17 years) • Text message-based survey: 541 (5 to 11 years with 2nd dose information)	• CVMS: 14 non-serious adverse events reported after the second vaccine dose (39.0 per 10,000 doses administered). These included redness and pain at the injection site, myalgia, fever, headache and chills • Text message-based survey: After the second dose, local adverse events were reported by 27.4% of respondents (148/541) and included pain, redness, swelling and itching at the injection site. Systemic adverse events included myalgia, headache or fatigue reported by 22.4% (121/541)
Malden 2022	• Electronic health record review (EHR) • self-reported digital survey data from the Kaiser Permanente Side Effect Monitor (KPSEM)	04 November 2021 to 28 February 2022	• KPSEM: 7,701; 6,247 completed survey responses received following first vaccine dose and 3,401 completed survey responses following the second vaccine dose • EHR, healthcare utilization for reported reactions: 129 following first dose and 59 following second dose • Medical record review conducted for 20 children whose parents sought medical care (emergency department visit or hospital admission) within 21 days following vaccination	• KPSEM: 33% (1,113/3,401) reported experiencing local injection site reactions. The most common reported local reaction was pain at the injection site. Systemic reactions reported by 32% (1,076/ 3,401); commonly reported symptoms included fatigue, headache, myalgia and fever • Local reactions were reported less frequently following the second dose 32.7% (1,113/3,401) than following the first dose 50.3% (3,140/6,247) • EHR showed that parents rarely sought care for post-vaccination symptoms, and among those seeking care, the most common symptoms documented in EHR were fever and nausea, comprising <0.5% of children. No encounters were related to myocarditis • Medical record review: 8 children identified for whom the hospital encounter was possibly associated with the vaccine; their diagnoses included non-specific nausea (n = 2), fever (n = 1), right lower quadrant pain (n = 1), gastroenteritis (n = 1), acute appendicitis (n = 1), allergy (n = 1), and abdominal pain (n = 1). Three (37.5%) of these eight patients reported chest pain or tachycardia via KPSEM in the 0–14 days following vaccination, but their medical charts did not contain evidence for myocarditis or pericarditis
Ripabelli 2022	Telephonic survey	December 2021 to January 2022	229 first vaccine dose recipients and 199 second vaccine dose recipients	• 84.3% (193/229) had at least one adverse event after the first dose and 73.4% (146/199) after the second vaccine dose • Local symptoms after the first and second dose occurred in 183 (94.8%) and 141 (96.6%) recipients (p = 0.435), respectively, while systemic reactions in 62 (32.1%) and 34 (23.3%) (p = 0.074) • Injection site pain, redness and swelling were commonly reported local adverse events whilst common systemic symptoms reported were lymphadenopathy, headache, fatigue, fever and chills
**STUDY ID**	**SURVEILLANCE SYSTEM USED**	**PERIOD**	**ADVERSE EVENTS REPORTED OR NUMBER OF CHILDREN RECEIVING TWO VACCINE DOSES (N)**	**FINDINGS**
Wood 2022	AusVaxSafety, online survey, active surveillance system	July 2021 to May 2022	211,994 survey responses for children aged 5–11 years, 79,542 responses from children who received two vaccines doses	• Adverse events were reported in 28% (22,115/79,542) of children following the second vaccine dose • Local adverse events such as injection site pain, itching, redness and swelling was reported in 23% (18,560/79,542) and systemic adverse events in 13% (10,234/79,542). The latter included myalgia/arthralgia, headache, fever and chills, fatigue and gastrointestinal symptoms • Medical review rates were low: 0.7% (570/79542) and impact on daily activities was also low: 7% (5,866/79542) • There were no self-reported cases of myocarditis or pericarditis
Yoshida 2022	Fukushima Vaccination Community Survey (FVCS)	02 April 2022 to 29 June 2022	421 children who had received two vaccine doses	• Local adverse events were common following the second vaccine dose; 73.9% (311/421) and included injection site pain • Systemic adverse events included fatigue (15.9%, 67/421), headache (14.3%, 60/421), fever (10.7%, 45/421), muscle/ joint pain (4.8%, 20/421) and nausea (3.3%, 14/421) • Among the 216 patients with allergic disease, 48 (22.2%) had experienced worsening of their chronic diseases • Having asthma was positively associated with experiencing systemic adverse events (OR, 2.24; 95% CI, 1.08–4.66)

Of note, we did not find studies that explored vaccine efficacy or effectiveness in high-risk children, including children with chronic respiratory disease, chronic heart conditions, chronic conditions of the kidney, liver or digestive system, chronic neurological disease, endocrine disorders, immunosuppression, asplenia or splenic dysfunction, or serious genetic abnormalities.

Since the initial review that informed the evidence-based recommendations by the SA National Department of Health [3], and following the last search in December 2022, we have identified three additional studies for consideration (See S3 Table). Jang *et al*. [63] aimed to evaluate the real-world effectiveness of BNT162b2 against COVID-19 infection in children during an Omicron-dominant period in South Korea. Khan *et al*. [64] conducted a test-negative case-control study in the US and Puerto Rico among children tested at a large US retail pharmacy chain. The third study by Tartof *et al*. [65] evaluated variant-specific (in the periods of both Delta and Omicron predominance) VE at the Kaiser Permanente Southern California (KPSC) health system with a matched test-negative case-control study. In all three studies, the two-dose 10μg dose of BNT162b2 was associated with modest protection against the Omicron variant. VE ranged from 20% (95% CI 17% to 23%) at four months [64] to 28% (95% CI 8% to 43%) after ≥3 months [65] and 41.2% (95% CI 34.3% to 47.4%) at 61–90 days [63]. Addition of these studies to the current rapid review is unlikely to change the direction or strength and certainty of recommendations in terms of effectiveness and safety of the two-dose BNT162b2 (Pfizer- BioNTech) COVID-19 vaccine in children aged 5 to 11 years.

## Discussion

The RCT by Walter *et al*. [18] found that two 10μg doses of the BNT162b2 vaccine is likely safe and 91% effective against COVID-19 infection in children aged 5 to 11 years (considered to be moderate certainty evidence). In absolute terms, this is 19 fewer cases per 1,000 vaccines delivered (ranging from 15 to 21 fewer cases). Similarly, observational studies found 65% effectiveness in preventing incident COVID-19 infection and 76% effectiveness in preventing hospitalization (moderate certainty evidence). The absolute effect is 167 fewer cases per 1,000 vaccines given (ranging from 130 fewer to 196 fewer cases) and 4 fewer hospitalisations per 10,000 children (from 3 fewer to 5 fewer hospitalisations). The differences in vaccine effectiveness across the studies may be explained by inherent issues of bias, residual confounding by behavioural factors such as mask-wearing or physical distancing, or by timing of vaccination, duration of follow-up in studies, dominant variants in the study period (i.e., Delta *vs*. Omicron), and testing policy changes (i.e., home/antigen testing *vs*. PCR-based tests) and if testing practices differed by vaccination status. Previous COVID-19 infection may influence vaccine effectiveness–unvaccinated children with previous infection may have infection-induced immunity that could bias vaccine effectiveness estimates toward the null, while vaccinated persons with previous infection may have higher levels of protection than those receiving vaccination alone [66].

Adverse events following vaccination with BNT162b2 were mild or moderate and transient. Injection-site pain was the most common local reaction, with fatigue and headache being the most frequently reported systemic events [18]. This finding was also reported across the included observational studies [44, 47, 54, 55, 57, 60–62]. The CDC passive vaccine safety surveillance system report identified serious adverse events such as seizures and myocarditis [44]. Similarly, in the CVMS passive vaccine safety surveillance system used in South Korea [60] there was one reported case of suspected myocarditis/pericarditis and one suspected case of anaphylaxis following the first vaccine dose. These cases were not medically confirmed diagnoses. However, in the absence of a comparator group this evidence was considered to be of very low certainty.

Most systematic reviews found assessed vaccine efficacy/effectiveness among adults and/or adolescents and children and evaluated other vaccines such as mRNA1273, Ad26.COV2 and CoronaVac in addition to BNT162b2 [67–75]. Sabu *et al*. [74] conducted a systematic review that evaluated the effectiveness of the BNT162b2 vaccine in children and adolescents. Findings were similar to those reported in this review; the two-dose BNT162b2 VE against COVID infection was 92% (95% CI 86% to 96%). Vaccination also offered protection against hospitalisation (91%) and intensive care admission (85%). Most adverse events reported were “transient and mild, commonly including pain at the injection site, fatigue and headache” [74].

In March 2023, the WHO’s Strategic Advisory Group of Experts on Immunization (SAGE) revised the roadmap for prioritizing the use of COVID-19 vaccines; outlining three priority-use groups: high, medium, and low [76]. Healthy children and adolescents aged 6 months to 17 years now form part of the low priority group. In this group, primary and booster doses are safe and effective, “however, considering the low burden of disease, SAGE urges countries considering vaccination of this age group to base their decisions on contextual factors, such as the disease burden, cost effectiveness, and other health or programmatic priorities and opportunity costs” [76].

Evidence informed decision-making involves the integration of the best available research evidence with clinical expertise and patient values [77]. Components of the evidence to decision framework to consider are resources and costs, feasibility, access, and acceptability [78]. Regarding feasibility and access to the BNT162b2 vaccine in children aged 5 to 11 years in SA, the paediatric-specific vaccine has been registered by SAHPRA in November 2022 [79]. It has been approved for use in “children with chronic cardiovascular, respiratory, neurological, endocrine, kidney, liver, or gastrointestinal conditions, as well as immunosuppression (including asplenia or dysfunction on the spleen) and serious genetic abnormalities affecting a number of systems” [7]. It is available as a dilute to use pediatric vaccine [79]. Additionally, the estimated cost of the paediatric version of the vaccine in South Africa is not yet available. In high income countries such as the United States and United Kingdom, COVID-19 vaccines were offered to children and adolescents as early as November 2021 [12, 80], however this has not been the case in Africa where inequities in access to vaccines exist, together with COVID-19 vaccine supply challenges and health system infrastructure issues [81]. These have impacted on the roll-out of vaccines resulting in few African countries modifying their vaccination strategies to include a wider range of children and adolescents aged 5 years and older [81].

Vaccination is considered a critically important public health intervention for reducing childhood morbidity and mortality worldwide. However, in 2021 approximately 18.1 million children globally did not receive their full series of basic immunizations, although this improved to 14.3 million in 2022 [82]. Reasons for poor vaccine coverage are multifactorial. One aspect is how different factors influence parental views and practices regarding routine childhood vaccinations [83]. Whilst COVID-19 vaccination does not form part of routine childhood vaccinations, parental attitudes, and perceptions play an important role in vaccine hesitancy and should be explored. The SA GRADE Network conducted a rapid search of available qualitative evidence regarding vaccination for 5 to 11-year olds. PubMed, Epistemonikos and Google Scholar databases were searched on 19 July 2022, and 8 August 2023. Seven qualitative studies were found, mostly from Europe and North America [84–90]; none of the studies were conducted in Africa. Parents felt that the risk of COVID-19 in children was low and, consequently, vaccination was not of high importance. Those parents with positive vaccination intentions were predominantly motivated by a desire to return to normality through reducing disease transmission by vaccinating their children thereby protecting their children and society in general. A recent meta-analysis of quantitative studies assessing COVID-19 vaccine hesitancy among parents in low- and middle-income countries [91], found parents not wanting to vaccinate their children had concerns about vaccine efficacy, safety and side effects. Of the 49% of parents willing to vaccinate their children, most believed that vaccination was crucial to ending the pandemic. This was further echoed by a systematic review and meta-analysis focusing on a worldwide estimation of parental acceptance of COVID-19 vaccines for their children [92] with approximately 57% of parents willing to vaccinate their children against COVID-19 –vaccine hesitancy among parents is present worldwide. In summary, across studies, most participants (usually parents or caregivers) were unlikely to vaccinate their children for COVID-19 with common reasons being concerns around vaccine safety and efficacy, side effects, and a feeling that it is not necessary. These findings may be applicable regardless of where parents are located in the world.

Acceptability of receiving the vaccine is likely to vary in SA, like in other countries. The uptake of vaccination amongst those aged 12–17 years in SA has been low, which may predict low uptake in the 5 to 11-year-old age group. According to Stats SA, the 2022 population estimate for 10-14-year-olds is 5,714,377 [93]. Among those aged 12 to 17 years, a total of 3,074,067 doses had been administered by 13 March 2023, with only 887,448 being second doses (~28.9% vaccine coverage) [94]. In the 5 to 11-year age group, parental consent would be required. Since this vaccination has only recently been approved in SA in this age group, we do not have local data on acceptability.

The strength of this review is that it used systematic review methods to rapidly evaluate the safety and efficacy of BNT162b2 COVID-19 vaccine in children to inform the national decision. The review team comprised both content and methods experts. We applied rigorous Cochrane methodology, risk of bias appraisal of studies and GRADE assessment of the quality of the evidence. There was a high degree of heterogeneity in the available data as noted in Figs 2 and 3, however, there is consistent agreement with the observational studies all indicating benefit. Considering the high heterogeneity observed, we have used various approaches to make sense of the data including random effects analysis, subgroup analyses by study design, checking that the studies were similar enough prior to pooling (the interventions, comparators and outcomes evaluated were similar across the pooled studies), and avoiding pooling RCTs and observational data. Published and ongoing studies were included in this review. Limitations were the challenge of including all relevant data, as the COVID-19 vaccination relevant publications emerge continuously and rapidly. However, for the decision process we were required to stop updated searches in order to finalise the review and inform the national decision. An example of this is the three observational studies that were published subsequent to finalisation of our review, however, their results confirmed our findings and did not add or deviate from the current evidence base [63–65]. Other limitations to the rapid review process include limiting the database search to three main databases, searching only for English language publications and inclusion of non-peer reviewed sources such as preprints [95]. Data in the current review informed the national decision and recommendations in SA and addition of data from these and other emerging studies is unlikely to change the findings substantively or direction of the recommendation.

## Conclusions

On balance, the available evidence demonstrated a small absolute benefit in prevention of incident COVID-19 cases and hospitalisation if a two-dose BNT162b2 vaccine is offered to children aged 5 to 11 years, compared to placebo (moderate certainty evidence). The availability and cost of the paediatric vaccine is yet to be confirmed. We did not find data on vaccine efficacy, safety and effectiveness in children aged 5 to 11 years with comorbidities. While there were no data to inform our understanding of vaccine effectiveness in this specific group of children, it is anticipated that children at greater risk of severe disease related to COVID-19 may have the greatest benefit from vaccination. This review formed part of the advisory to the Ministry of Health in SA which recommends use of the two dose COVID-19 vaccination for children aged 5 to 11 years with comorbidities guided by their healthcare provider. The National Advisory has been published online [3, 7] and recommendations are being actioned for national implementation. These recommendations are in line with current WHO guidance [76]. Further studies are needed to evaluate long-lasting immunity in children, including the need for booster doses. Specific studies assessing vaccine safety and efficacy in children with comorbidities are needed. Qualitative studies on parental and children’s preferences are needed to determine acceptability of COVID-19 vaccination in five to 11-year-olds to inform contextually relevant decisions globally.

## Supporting information

S1 Checklist PRISMA checklist. (DOCX)

S1 Table Search terms and strategy. (DOCX)

S2 Table Characteristics of included studies. (DOCX)

S3 Table Characteristics of planned and ongoing studies. (DOCX)

S4 Table Characteristics of excluded studies. (DOCX)

S1 Fig ROBINS-I assessments for observational studies. (TIF)
