# Supplementary material for: Effectiveness of the BNT162b2 vaccine in preventing morbidity and mortality associated with COVID-19 in children aged 5 to 11 years: A systematic review and meta-analysis
Source: PLOS Glob Public Health. 2023 Dec 4;3(12):e0002676. doi: 10.1371/journal.pgph.0002676 (PMC10695397; doi:10.1371/journal.pgph.0002676)
Supplement: S1 Table — (DOCX) [file pgph.0002676.s002.docx]

**S1 TABLE. SEARCH TERMS AND STRATEGY**

| **Living Review: McMaster Vaccine Effectiveness Review [23 June 2022]**  ***Output****:* <https://www.mcmasterforum.org/docs/default-source/product-documents/living-evidence-syntheses/covid-19-living-evidence-synthesis-8.13---what-is-the-effectiveness-of-available-covid-19-vaccines-for-children-and-adolescents-including-variants-of-concern.pdf?sfvrsn=2428374_7> |
| --- |
| **Cochrane COVID-19 Study Register [23 June 2022]**  **Search strategy**: Pfizer or BNT162b2 or BioNtech or Comirnaty  **Output**: 3805 studies with 4467 references (IMPORTED 4463 into EndNote) Filtered EndNote results  Title – word begins with CHILD OR  Abstract – word begins with CHILD  Abstract – word begins with YOUNG NOT  Abstract – contains WOMAN OR WOMEN OR ADULT OR PATIENTS OR WORK OR MALE OR MEN  175 studies |
| **COVID-nma [27 June 2022] (https://covid-nma.com/)**  ***Search terms/ strategy:*** *Living Evidence Synthesis (Vaccine RCTs) -> Filter by author “Walter”* ***Output:*** <https://covid-nma.com/vaccines/index.php?search_by=2&search_input=walter&submit=Validate#moteur_recherche> (n=1) |
| **Living Review: McMaster Vaccine Effectiveness Review [29 November2022]**  ***Output****:* <https://www.mcmasterforum.org/docs/default-source/product-documents/living-evidence-syntheses/covid-19-living-evidence-synthesis-8.18---what-is-the-effectiveness-of-available-covid-19-vaccines-for-children-and-adolescents-including-variants-of-concern.pdf> |
| **Cochrane COVID-19 Study Register [7 December 2022]**  **Search strategy:** (Pfizer or BNT162b2 or BioNtech or Comirnaty) and child*  **Records retrieved:** 220 studies |
