# Supplementary material for: Effectiveness of the BNT162b2 vaccine in preventing morbidity and mortality associated with COVID-19 in children aged 5 to 11 years: A systematic review and meta-analysis
Source: PLOS Glob Public Health. 2023 Dec 4;3(12):e0002676. doi: 10.1371/journal.pgph.0002676 (PMC10695397; doi:10.1371/journal.pgph.0002676)
Supplement: S2 Table — (DOCX) [file pgph.0002676.s003.docx]

**S2 TABLE. CHARACTERISTICS OF INCLUDED STUDIES**

| **CITATION** | **STUDY DESIGN** | **POPULATION (N)** | **TREATMENT** | **OUTCOMES** | **RISK OF BIAS** |
| --- | --- | --- | --- | --- | --- |
| **RANDOMISED CLINICAL TRIAL** | | | | | |
| Walter EB, Talaat KR, Sabharwal C, Gurtman A, Lockhart S, Paulsen GC, *et al*. Evaluation of the BNT162b2 covid-19 vaccine in children 5 to 11 years of age. N Engl J Med. 2022; 368:35-46. | Setting and design  Phase 1 dose-level identification study and Phase 2 – 3 safety, immunogenicity and efficacy double-blind randomised controlled trial across 81 sites in the United States, Spain, Finland and Poland  Participants were randomly assigned (interactive web-based system) 2:1 ratio to receive 2 doses, 21 days apart.  Dominant Variant(s)  Delta  Follow‐up duration (days)  30-90 | Sample size  *Phase 2-3 trial*  Healthy children aged 5 to 11 years (N = 2,285 were randomised; Intervention n= 1,528, Control n= 757)  The number that received injections was n = 2,268, 1,517 to Intervention, 751 to Control. One patient erroneously got vaccine instead of placebo, so numbers were 1,518 and 750 respectively. | Intervention  BNT162b2 vaccine (10µg), 2 doses, 21 days apart  Control  Saline placebo, 2 doses, 21 days apart | Primary outcome(s)   - Safety/Adverse events - Vaccine Efficacy: Estimated efficacy of vaccine in preventing SARS-CoV-2 infection | Overall: Some concerns of bias  Randomisation: low risk of bias  Deviations from the intervention: some risk of bias  Missing outcome data: low risk of bias  Measurement of the outcome: low risk of bias  Selection of the reported results: low risk of bias |
| **OBSERVATIONAL STUDIES** | | | | | |
| Amir O, Goldberg Y, Mandel M, Bar-On YM, Bodenheimer O, Freedman L, Ash N, Alroy-Preis S, Huppert A, Milo R. Initial protection against SARS-CoV-2 omicron lineage infection in children and adolescents by BNT162b2 in Israel: an observational study. The Lancet Infectious Diseases. 2023 Jan 1; 23(1):67-73. | Setting and Design  Prospective cohort study in Israel. Study period: December 26, 2021, through  January 8, 2022  Dominant Variant/s  Omicron  Follow‐up duration (days)  14-35 | Sample size  N = 701,381 children aged 5-10 years  Children in the 5-10 age group were divided into three cohorts: those unvaccinated, those who  received the second dose of vaccine at least 14 days previously, and an internal control cohort of those who received their first dose 3-7 days previously | Intervention  BNT162b2 (two doses) received at least 14 days before  Control  Unvaccinated and an internal control cohort of those who received their first dose 3-7 days previously | Primary outcome(s)  Adjusted rates of confirmed infection following two doses of the BNT162b2 vaccine in children aged 5-10 up to 35 days from the 2nd dose | ROBINS-I: Moderate risk of bias  Downgraded due to study design and accounting for non-immune period and positive  Study design – moderate risk – data-linkage.  The authors specified the non-immune period and checked for an effect but there WAS an effect (e.g., evidence of a reduction in an outcome of interest when there should not have been) = evidence of residual bias |
| Bloise S, Marcellino A, Frasacco B, Gizzone P, Proietti Ciolli C, Martucci V, Sanseviero M, Del Giudice E, Ventriglia F, Lubrano R. Cross-Sectional Survey on BNT162b2 mRNA COVID-19 Vaccine Serious Adverse Events in Children 5 to 11 Years of Age: A Monocentric Experience. Vaccines. 2022 Jul 30; 10(8):1224. | Setting and Design  Cross-sectional study in Italy. Study period: December 15, 2021, through  January 11, 2022  Dominant Variant/s  Omicron  Follow‐up duration (days)  1-20 | Sample size  N = 569 children 5-11 years old who received at least one vaccine dose | Intervention  BNT162b2 (one or two doses)  Control  Nil | Primary outcome(s)  Adverse events after the first and second doses | ROBINS-I: Serious risk of bias  Downgraded due to study design, previously infected not excluded, no information available for many domains |
| Capponi M, Pulvirenti F, Cinicola BL, Brindisi G, Conti MG, Colaiocco G, de Castro G, Guido CA, Duse M, Midulla F, Zicari AM. Short-term side effects and SARS-CoV-2 infection after COVID-19 pfizer–BioNTech vaccine in children aged 5–11 years: An Italian real-world study. Vaccines. 2022 Jun 30; 10(7):1056. | Setting and Design  Cross-sectional study in Italy. Study period: February to March 2022  Dominant Variant/s  Omicron  Follow‐up duration (days)  30 | Sample size  N = 579 children 5-11 years old who received at least one vaccine dose | Intervention  BNT162b2 (one or two doses)  Control  Nil | Primary outcome(s)  Adverse events after the first and second dose | ROBINS-I: Serious risk of bias  Downgraded due to study design, previously infected not excluded, no information available for many domains |
| Chemaitelly H, AlMukdad S, Ayoub HH, Altarawneh HN, Coyle P, Tang P, Yassine HM, Al-Khatib HA, Smatti MK, Hasan MR, Al-Kanaani Z. COVID-19 vaccine protection among children and adolescents in Qatar. New England Journal of Medicine. 2022 Nov 17; 387(20):1865-76. | Setting and Design  Retrospective, matched cohort study emulating randomized target trials, conducted in Qatar between Feb 03, 2022 and July 12, 2022  Dominant Variant/s  Omicron  Follow‐up duration (days)  14-110 | Sample size  N = 37,456 children aged 5-11 years  18,728 vaccinated children and 18,728 unvaccinated matched controls | Intervention  BNT162b2 (two doses) received at least 14 days before  Control  Unvaccinated control cohort | Primary outcome(s)   - Documented SARS-CoV-2 infection: A PCR or rapid antigen confirmed infection - Real world effectiveness of the two-dose primary vaccine series against severe SARS-CoV-2 | ROBINS-I: Moderate Risk of bias  Downgraded due to study design, cohort with no concerns |
| Cocchio S, Zabeo F, Tremolada G, Facchin G, Venturato G, Marcon T, Saia M, Tonon M, Mongillo M, Da Re F, Russo F. COVID-19 vaccine effectiveness against Omicron variant among underage subjects: the Veneto region’s experience. Vaccines. 2022 Aug 20; 10(8):1362. | Setting and Design  Data linkage study in Veneto, Italy; during two time periods Aug 01 - Oct 21, 2021 and Feb 01 - Apr 27, 2022  Dominant Variant/s  Delta and Omicron  Follow‐up duration (days)  ≥70 | Sample size  N = 193,509 children aged 5-11 years  71,296 unvaccinated children in the Delta period (2021). 81,895 unvaccinated children, 17,632 children who received one vaccine dose and 22,686 children who received two doses in the Omicron period (2022) | Intervention  Partial – one dose BNT162b2  Fully vaccinated – two doses BNT162b2  Control  Unvaccinated children | Primary outcome(s)  Documented SARS-CoV-2 infection: A PCR or rapid antigen confirmed infection | ROBINS-I: Serious risk of bias  Downgraded due to accounting for non- immune period and positive |
| Cohen-Stavi CJ, Magen O, Barda N, Yaron S, Peretz A, Netzer D, Giaquinto C, Judd A, Leibovici L, Hernán MA, Lipsitch M. BNT162b2 Vaccine Effectiveness against Omicron in Children 5 to 11 Years of Age. New England Journal of Medicine. 2022 Jun 29. | Setting and design  Data linkage study conducted in Israel. Study period: Nov 23, 2021 to January 7, 2022  Dominant Variant/s  Omicron  Follow‐up duration (days)  42 | Sample size  N= 189,456  94,728 vaccinated children and 94,728 unvaccinated matched controls | Intervention  BNT162b2 (two doses)  Control  Unvaccinated children | Primary outcome(s)   - Documented SARS-CoV-2 infection: A PCR confirmed infection - Symptomatic COVID-19 | ROBINS-I: Serious risk of bias  Downgraded because authors did not reported methods for confirming vaccination but it is highly suspected that they used a linked database |
| Dorabawila V, Hoefer D, Bauer UE, Bassett M, Lutterloh E, Rosenberg E. Effectiveness of the BNT162b2 vaccine among children 5-11 and 12-17 years in New York after the Emergence of the Omicron Variant. MedRxiv. 2022 Jan 1. | Setting and Design  Data-linkage study in New York state, USA. Study period: December, 2021- Jan, 2022  Dominant Variant/s  Omicron  Follow‐up duration (days)  48 | Sample size  N = 365,502 children aged 5-11 years were fully vaccinated | Intervention  BNT162b2 (two doses) received at least 14 days before  Control  Unvaccinated children | Primary outcome(s)   - Laboratory confirmed COVID-19 cases, defined as positive Nucleic Acid Amplification Test (NAAT) or antigen results - New COVID-19 hospital admissions | ROBINS-I: Serious risk of bias  Downgraded as there was no adjustment for comorbidities, previously infected were not excluded |
| Fleming-Dutra KE, Britton A, Shang N, Derado G, Link-Gelles R, Accorsi EK, Smith ZR, Miller J, Verani JR, Schrag SJ. Association of Prior BNT162b2 COVID-19 Vaccination With Symptomatic SARS-CoV-2 Infection in Children and Adolescents During Omicron Predominance. JAMA. 2022 May 13. | Setting and Design  Test-negative case-control design in 49 states of the U.S among persons aged 5–15 years with COVID-19–like illness during Dec 26, 2021– Feb 21, 2022, including 74,208 tests from children 5 to 11 years of age and 47,744 tests from adolescents 12 to 15 years of age  Dominant Variant/s  Omicron  Follow‐up duration (days)  90 | Sample size  N= 74,208 children aged 5-11 years in the analytic  data set: 58,430 unvaccinated and 15,778 vaccinated with two doses | Intervention  BNT162b2 (two doses)  Control  Unvaccinated children | Primary outcome(s)  Symptomatic SARS-CoV-2 infection | ROBINS-I: Serious risk of bias  Downgraded because vaccination status was assessed by questionnaire without confirmation by an additional method |
| Fowlkes AL, Yoon SK, Lutrick K, Gwynn L, Burns J, Grant L, Phillips AL, Ellingson K, Ferraris MV, LeClair LB, Mathenge C. Effectiveness of 2-dose BNT162b2 (Pfizer BioNTech) mRNA vaccine in preventing SARS-CoV-2 Infection among children aged 5–11 years and adolescents aged 12–15 years—PROTECT Cohort, July 2021–February 2022. Morbidity and Mortality Weekly Report. 2022 Mar 18; 71(11):422. | Setting and Design  Prospective cohort in four states of US (Arizona, Florida, Texas, and Utah), of 1,364 participants between Jul 2021–Feb 2022; the PROTECT cohort  Dominant Variant/s  Omicron  Follow‐up duration (days)  82 | Sample size  N = 1,052 children aged 5-11 years  301 unvaccinated and 751 vaccinated with ≥1 vaccine dose (682 received 2 doses and 69 received 1 dose) | Intervention  BNT162b2 (two doses)  Control  Unvaccinated children | Primary outcome(s)  Symptomatic and asymptomatic SARS-CoV-2 infections | ROBINS-I: Moderate risk of bias  Downgraded due to study design, and because parents or legal guardians provided the participants’ vaccination history confirmed by immunization registries |
| Hause AM, Baggs J, Marquez P, Myers TR, Gee J, Su JR, Zhang B, Thompson D, Shimabukuro TT, Shay DK. COVID-19 vaccine safety in children aged 5–11 years—United States, November 3–December 19, 2021. Morbidity and Mortality Weekly Report. 2021 Dec 12; 70(51-52):1755. | Setting and Design  Data linkage study. A Vaccine Adverse Event Reporting System (VAERS), a passive vaccine safety surveillance system, and adverse events and health impact assessments) November 3–December 19, 2021 in the US  Dominant Variant/s  Omicron  Follow‐up duration (days)  82 | Sample size  N = 4,249 reports of adverse events: VAERS  N = 29,899 children with 2^nd^ dose information for v-safe | Intervention  BNT162b2 (two doses)  Control  Nil | Primary outcome(s)   - Adverse events - Health impact assessments | ROBINS-I: Serious risk of bias  Downgraded due to study design, no information available for many domains  Cohort with any concerns = serious (passive surveillance, subject to reporting biases and underreporting) |
| Kim S, Heo Y, Seo SY, Lim DS, Cho E, Lee YK. Adverse events of the Pfizer-BioNTech COVID-19 vaccine in Korean children and adolescents aged 5 to 17 years. Osong Public Health and Research Perspectives. 2022 Oct 14; 13(5):382-90. | Setting and Design  Data linkage study. COVID-19 vaccination management system (CVMS, a web-based passive vaccine safety surveillance system) and text message-based safety surveillance system in South Korea. Study period: March 05, 2021 to July 20, 2022  Dominant Variant/s  Omicron  Follow‐up duration (days)  0-7 | Sample size  N = 94,518 children 5-11 years old who received at least one vaccine dose | Intervention  BNT162b2 (one or two doses)  Control  Nil | Primary outcome(s)  Adverse events | ROBINS-I: Serious risk of bias  Downgraded due to study design, no information available for many domains  Cohort with any concerns = serious (passive surveillance, subject to reporting biases and underreporting) |
| Klein NP, Stockwell MS, Demarco M, Gaglani M, Kharbanda AB, Irving SA, *et al*. Effectiveness of COVID-19 Pfizer-BioNTech BNT162b2 mRNA vaccination in preventing COVID-19-associated emergency department and urgent care encounters and hospitalizations among non-immunocompromised children and adolescents age 5 to 17 years – VISION network, 10 states, April 2021 – January 2022. MMWR. 2022; 71(9):352-8. | Setting and design:  A case-control test-negative design of 39,217 emergency department (ED) and urgent care (UC) encounters and 1699 hospitalizations between children and adolescents aged 5 to 17 years with COVID-19 like illness, across ten states in the United States (US) between 9 April 2021 to 29 January 2022  Dominant variant(s) Omicron and Delta  Follow‐up duration (days)  14-67 | Sample size  N = 9,181 (Unvaccinated n = 8,599; two doses Pfizer-BioNTech [14 to 149 days earlier] n = 582) | Intervention  BNT162b2 (two doses)  Control  Unvaccinated children | Primary outcome(s)  Estimated vaccine effectiveness (VE) in ED/UC encounters and hospitalisation | ROBINS-I: Serious risk of bias  Downgraded because the index date for each medical event was defined as the date of the most recent positive or negative result prior to the medical event (14 days before admission) OR the date of the medical event |
| Lin D, Gu Y, Xu Y, Zeng D, Wheeler B, Young H, Sunny SK, Moore Z. Effects of vaccination and previous infection on Omicron infections in children. New England Journal of Medicine. 2022 Sep 22; 387(12):1141-3. | Setting and Design  Prospective cohort study conducted in North Carolina, USA between Dec 2021 and June 2022  Dominant Variant/s  Omicron  Follow‐up duration (days)  14-88 | Sample size  N = 887,193 children aged 5-11 years  614,036 unvaccinated children, 37,759 children who received one vaccine dose and 228,123 children who received two doses and 7,275 children who received a booster (third vaccine dose) | Intervention  Partial – one dose BNT162b2  Fully vaccinated – two doses BNT162b2  Control  Unvaccinated children | Primary outcome(s)   - SARS-CoV-2 infections (the incidence of notified SARS-CoV-2 infection (asymptomatic or symptomatic) - Severe COVID-19 (defined as a SARS-CoV-2 infection resulting in hospital admission or death) | ROBINS-I: Moderate risk of bias  Downgraded due to study design, cohort with no concerns |
| Malden DE, Gee J, Glenn S, Li Z, Mercado C, Ogun OA, Kim S, Lewin BJ, Ackerson BK, Jazwa A, Weintraub ES. Reactions following Pfizer-BioNTech COVID-19 mRNA vaccination and related healthcare encounters among 7,077 children aged 5-11 years within an integrated healthcare system. Vaccine. 2023 Jan 9; 41(2):315-22. | Setting and Design  Data linkage study. The Kaiser Permanente Side  Effect Monitor (KPSEM) and EHR-based healthcare utilization data from Kaiser Permanente Southern California (KPSC), US. Study period: Nov 2021 to March 2022  Dominant Variant/s  Omicron  Follow‐up duration (days)  1-35 | Sample size  N = 7,077 children 5-11 years old who received at least one vaccine dose | Intervention  BNT162b2 (one or two doses)  Control  Nil | Primary outcome(s)  Adverse events | ROBINS-I: Serious risk of bias  Downgraded due to study design, no information available for many domains  Cohort with any concerns = serious (passive surveillance, subject to reporting biases and underreporting) |
| Piché-Renaud PP, Swayze S, Buchan S, Wilson S, Austin PC, Morris SK, Nasreen S, Schwartz KL, Tadrous M, Thampi N, Wilson K. Vaccine Effectiveness of BNT162b2 Against Omicron in Children Aged 5-11 Years: A Test-Negative Design. | Setting and Design  Test-negative case-control design in Ontario, Canada among persons aged 5–11 years with COVID-19–like illness during January 02, 2022– May 28, 2022, including 5,870 test-positive cases and 7,050 test-negative controls  Dominant Variant/s  Omicron  Follow‐up duration (days)  ≥60 | N= 12,920 (Unvaccinated n = 4,314; one dose received n = 3,587; two doses Pfizer-BioNTech [8-week dosing interval] n = 5,019) | Intervention  Partial – 1 dose BNT162b2  Fully vaccinated – two doses BNT162b2  Control  Unvaccinated children | Primary outcome(s)   - VE against symptomatic infection - VE against severe outcomes (death or hospitalization) | ROBINS-I: Serious risk of bias  Downgraded because symptoms were verified by using sample date without interview or documented confirmation of symptoms ≤ 10 days (relevant for symptomatic disease only) |
| Price AM, Olson SM, Newhams MM, Halasa NB, Boom JA, Sahni LC, *et al*. BNT162b2 protection against the Omicron variant in children and adolescents. N Engl J Med. 2022; 386 (2):1899:090. | Setting and Design  A case-control, test-negative design at 31 hospitals across 23 states in the US between 1 July 2021 and 17 February 2022.  Dominant Variant(s)  Omicron-predominant period  Follow‐up duration (days)  14-88 | Sample size  N = 537 (cases n = 267; controls n = 270) | Intervention  BNT162b2 (two doses) received at least 14 days before  Control  Unvaccinated | Primary outcome(s)   - Vaccine effectiveness against COVID-19–associated hospitalization - Vaccine effectiveness against a gradient of disease severity | ROBINS-I: Serious Risk of bias  Downgraded to serious as the authors didn't excluded or analyse separately participants with prior COVID-19 infection (concerns about infectivity and risk-taking/health-seeking behavior) |
| Ripabelli G, Sammarco ML, D’Amico A, De Dona R, Iafigliola M, Parente A, Samprati N, Santagata A, Adesso C, Natale A, Di Palma MA. Safety of mRNA BNT162b2 COVID-19 (Pfizer-BioNtech) vaccine in children aged 5–11 years: Results from an active pharmacovigilance study in central Italy. Human Vaccines & Immunotherapeutics. 2022 Nov 30; 18(6):2126668. | Setting and Design  Cross-sectional study in Italy. Study period: December 2021 to February 2022  Dominant Variant/s  Omicron  Follow‐up duration (days)  7-10 | Sample size  N = 229 children 5-11 years old who received at least one vaccine dose | Intervention  BNT162b2 (one or two doses)  Control  Nil | Primary outcome(s)  Adverse events after the first and second dose | ROBINS-I: Serious risk of bias  Downgraded due to study design, previously infected not excluded, no information available for many domains |
| Sacco C, Manso M Del, Mateo-Urdiales A, Rota MC, Petrone D, Riccardo F, *et al*. Articles Effectiveness of BNT162b2 vaccine against SARS-CoV-2 infection and severe COVID-19 in children aged 5-11 years in Italy: a retrospective analysis of January-April, 2022. Lancet 2022;400:97–103. | Setting and Design  Data linkage study. Study period: Jan 17, to April 13, 2022  Dominant variant  Omicron  Follow-up duration (days)  84 | Sample size  N = 2,965,918 (n = 1,063,035 two doses, n = 134,386 one dose, and n = 1,768,497 unvaccinated) | Intervention  Partial – one dose BNT162b2  Fully vaccinated – two doses BNT162b2  Control  Unvaccinated | Primary outcome(s)   - SARS-CoV-2 infections (the incidence of notified SARS-CoV-2 infection (asymptomatic or symptomatic) - Severe COVID-19 (defined as a SARS-CoV-2 infection resulting in hospital admission or death within 28 days) | ROBINS-I: Moderate risk of bias  Downgraded due to study design; data-linkage study and the authors specified the non-immune period and checked for an effect but there was an effect (e.g., evidence of a reduction in an outcome of interest when there should not have been) = evidence of residual bias (≥7 days) |
| Shi DS, Whitaker M, Marks KJ, Anglin O, Milucky J, Patel K, *et al*. Hospitalizations of children aged 5 – 11 years with laboratory-confirmed COVID-19 – COVID-NET, 14 states, March 2020 – February 2022. MMWR. 2022; 71(16):574:81. | Setting and Design  Data- linkage study -hospitalisation rates in children aged 5 to 11 years between 1 March 2020 and 28 February 2022with focus on the period of early Omicron predominance (19 December 2021 to 28 February 2022).  Dominant Variant(s)  Pre-Delta to Delta to Omicron  Follow‐up duration (days)  60 | Sample size  N = 1,475 (pre-Delta n = 596, Delta predominant n = 482, Omicron predominant n = 397) | Intervention  BNT162b2 (two doses) received at least 14 days before  Control  Unvaccinated | Primary outcome(s)   - Hospitalisation rates in the pre-Delta, Delta predominant and Omicron predominant waves - Length of hospital stay - Requirement of ICU admission - Non-invasive or invasive ventilation | ROBINS-I: Serious risk of bias  Downgraded due to study design, calendar time not adjusted for (accounting for calendar time reduces bias due to differences in vaccine accessibility and risk of exposure over time), previously infected not excluded |
| Simmons AE, Amoako A, Grima AA, Murison KR, Tuite AR, Fisman DN. Vaccine Effectiveness Against Hospitalization Among Adolescent and  Pediatric SARS-CoV-2 Cases in Ontario, Canada [Internet]. MedRxiv [Preprint]. 2022 [cited 29 Jun 2022]. | Setting and Design  Age and time-matched nested case-control design between 28 May 2021 and 10 January 2022 in 1,441 paediatric and adolescent patients aged 4 to 17 years old.  Dominant Variant(s)  Delta to Omicron  Follow‐up duration (days)  Not stated | Sample size  N = 753 children aged 4-11 years | Intervention  BNT162b2 (two doses) received at least 14 days before  Control  Unvaccinated | Primary outcome(s)  Hospitalisation due to SARS-CoV-2 | ROBINS-I: Serious risk of bias  Downgraded because there is no clarity about inclusion of participants with prior COVID-19 infection |
| Tan SH, Cook AR, Heng D, Ong B, Lye DC, Tan KB. Effectiveness of BNT162b2 Vaccine against Omicron in Children 5 to 11 Years of Age. New England Journal of Medicine. 2022 Jul 20. | Setting and Design  Data linkage study; a retrospective population analysis based on official data reported to and maintained by the Ministry of Health,  Singapore. Study period: January 21, 2022, through April 8, 2022  Dominant variant  Omicron  Follow-up duration (days)  Not stated | Sample size  N = 255,936 (n = 173,237 two doses, n= 30,656 partially vaccinated*, and n= 52,043 unvaccinated) | Intervention  Partial – on the day after they received the first dose up to 6 days after they received the second dose of BNT162b2  Fully vaccinated – 7 days or more after they received the second dose of BNT162b2  Control  Unvaccinated | Primary outcome(s)   - Laboratory confirmed SARS-COV-2 infection - Hospitalisation - Vaccine effectiveness | ROBINS-I: Serious risk of bias  Downgraded due to study design and authors did not adjust for comorbidities |
| Wood N, Lopez LK, Glover C, Leeb A, Cashman P, Deng L, Macartney K. Active safety surveillance of COVID-19 mRNA vaccines in children aged 5-15 years in Australia. medRxiv. 2022 Jul 22:2022-07. | Setting and Design  Data linkage study. AusVaxSafety (Australia’s active safety surveillance system). Study period: July 2021 to May 2022  Dominant Variant/s  Omicron  Follow‐up duration (days)  0-3 | Sample size  N = 211,994 survey responses in children aged 5-11 years | Intervention  BNT162b2 (one, two or three doses)  Control  Nil | Primary outcome(s)  Adverse events | ROBINS-I: Serious risk of bias  Downgraded due to study design, no information available for many domains |
| Yoshida M, Kobashi Y, Shimazu Y, Saito H, Yamamoto C, Kawamura T, Wakui M, Takahashi K, Ito N, Nishikawa Y, Zhao T. Time course of adverse reactions following BNT162b2 vaccination in healthy and allergic disease individuals aged 5–11 years and comparison with individuals aged 12–15 years: an observational and historical cohort study. European Journal of Pediatrics. 2023 Jan; 182(1):123-33. | Setting and Design  Observational cohort study based on the Fukushima Vaccination Community Survey (FVCS), Japan. Study period: April2, 2022 to June 29, 2022  Dominant Variant/s  Omicron  Follow‐up duration (days)  0-7 | Sample size  N = 421 children aged 5-11 years who received two vaccine doses | Intervention  BNT162b2 (two doses)  Control  Nil | Primary outcome(s)  Adverse events | ROBINS-I: Serious risk of bias  Downgraded due to study design, no information available for many domains |
| Zambrano LD, Newhams MM, Olson SM, Halasa NB, Price AM, Orzel AO, Young CC, Boom JA, Sahni LC, Maddux AB, Bline KE. BNT162b2 mRNA Vaccination Against COVID-19 is Associated with Decreased Likelihood of Multisystem Inflammatory Syndrome in US Children (MIS-C) Ages 5–18 Years. Clinical infectious diseases: an official publication of the Infectious Diseases Society of America. 2022 Aug 4. | Setting and Design  Test-negative case-control design in 29 hospitals in 22 states of U.S among hospitalized patients aged 5–18 years. Study period: Jul 1, 2021–Apr 7, 2022  Dominant Variant(s)  Delta to Omicron  Follow‐up duration (days)  28+ days | Sample size  N = 374 children aged 5-11 years  (Multisystem Inflammatory Syndrome in Children [MIS-C] cases n = 144; controls n = 230) | Intervention  BNT162b2 (two doses) received at least 14 days before  Control  Unvaccinated | Primary outcome(s)   - Vaccine effectiveness against MIS-C - Organ system involvement and critical disease in vaccinated versus unvaccinated patients with MIS-C | ROBINS-I: Serious Risk of bias  Downgraded to serious as the authors didn't excluded or analyse separately participants with prior COVID-19 infection (concerns about infectivity and risk-taking/health-seeking behavior) |
