# Supplementary material for: Effectiveness of the BNT162b2 vaccine in preventing morbidity and mortality associated with COVID-19 in children aged 5 to 11 years: A systematic review and meta-analysis
Source: PLOS Glob Public Health. 2023 Dec 4;3(12):e0002676. doi: 10.1371/journal.pgph.0002676 (PMC10695397; doi:10.1371/journal.pgph.0002676)
Supplement: S3 Table — (DOCX) [file pgph.0002676.s004.docx]

**S3 TABLE. CHARACTERISTICS OF PLANNED AND ONGOING STUDIES**

| **STUDY** | **SAMPLE SIZE** | **SEVERITY AT ENROLLMENT** | **SPONSOR** | **REGISTRATION NUMBER** | **FULL-TEXT LINK** | **SOURCE** |
| --- | --- | --- | --- | --- | --- | --- |
| A Phase 1/2/3 Study to Evaluate the Safety, Tolerability, and Immunogenicity of an RNA Vaccine Candidate Against COVID-19 in Healthy Children <12 Years of Age | 4,644 | Healthy volunteers | BioNTech SE | EUCTR2020-005442-42-PL | <https://www.clinicaltrialsregister.eu/ctr-search/trial/2020-005442-42/PL> | COVID-nma website ([www.covid-nma.com](http://www.covid-nma.com))  5 July 2022 |
| A Phase 1/2/3 Study to Evaluate the Safety, Tolerability, and Immunogenicity of an RNA Vaccine Candidate Against COVID-19 in Healthy Children and Young Adults | 11,111 | Healthy children and young adults | BioNTech SE | NCT04816643 | <https://clinicaltrials.gov/ct2/show/NCT04816643> | Cochrane COVID-19 register (<https://covid-19.cochrane.org/>)  28 June 2022 |
| National Vaccine, Institute. Safety and Immunogenicity of SARS-CoV-2 mRNA vaccine platform in Thai children aged 5-11 years | 400 | Healthy children | National Vaccine Institute | TCTR20220125002 | <https://trialsearch.who.int/Trial2.aspx?TrialID=TCTR20220125002> | Cochrane COVID-19 register (<https://covid-19.cochrane.org/>)  28 June 2022 |
| A Study to Learn About COVID-19 Bivalent BNT162b2 Omicron Containing Vaccine in Healthy Children | 6,550 | Healthy children | BioNTech SE | NCT05543616 | <https://clinicaltrials.gov/show/NCT05543616> | Cochrane COVID-19 register (<https://covid-19.cochrane.org/>)  07 December 2022 |
| Comparison of the safety and immunogenicity of two-dose mRNA COVID-19 vaccine and inactivated followed by an mRNA vaccine in children aged 5 - 11 years | 120 | Healthy children | Chulalongkorn University | TCTR20220212001 | <https://www.thaiclinicaltrials.org/show/TCTR20220212001> | Cochrane COVID-19 register (<https://covid-19.cochrane.org/>)  07 December 2022 |
| A retrospective cohort study of the safety and persistence of immunity to SARS-CoV-2 vaccine in children in Izunokuni | 1,700 | Healthy children aged 5-11 years living in Izu no Kuni City, Shizuoka | Juntendo University School of Medicine | JPRN-UMIN000046980 | <https://trialsearch.who.int/Trial2.aspx?TrialID=JPRN-UMIN000046980> | Cochrane COVID-19 register (<https://covid-19.cochrane.org/>)  07 December 2022 |
| Immune Persistence and Safety Survey (Pediatric Cohort Survey) related to the SARS-CoV-2 Vaccination (First and Second Vaccination) in Japan (Children Aged 5-11 Years Old) | 500 | Healthy children | Juntendo University School of Medicine | JPRN-UMIN000047368 | <https://trialsearch.who.int/Trial2.aspx?TrialID=JPRN-UMIN000047368> | Cochrane COVID-19 register (<https://covid-19.cochrane.org/>)  07 December 2022 |
| Immunogenicity and safety of the booster dose with SARS-CoV-2 mRNA vaccine following fully immunized with inactivated vaccine in Thai children aged 5-11 years for COVID-19 prevention | 60 | Healthy children | National Vaccine Institute | TCTR20220330001 | <https://trialsearch.who.int/Trial2.aspx?TrialID=TCTR20220330001> | Cochrane COVID-19 register (<https://covid-19.cochrane.org/>)  07 December 2022 |
| Phase 1/2 double-blinded study to Evaluate Adverse Events and Antibody level, Cell-Mediated Immune Response and Immune Response Against SAR-CoV-2 Variants after COVID-19 Vaccination in Thai Children and Adolescents | 800 | Healthy Thai children and adolescents | Program Management Unit for Competitiveness (PMUC) | TCTR20220406002 | <https://trialsearch.who.int/Trial2.aspx?TrialID=TCTR20220406002> | Cochrane COVID-19 register (<https://covid-19.cochrane.org/>)  07 December 2022 |
| Long term immunogenicity of BNT162b2 vaccination and attitudes toward COVID-19 booster vaccines in Thai children | 240 | Healthy children | Ratchadapisek Research Funds | TCTR20220301002 | <https://trialsearch.who.int/Trial2.aspx?TrialID=TCTR20220301002> | Cochrane COVID-19 register (<https://covid-19.cochrane.org/>)  07 December 2022 |
| **STUDIES UNDER CONSIDERATION** | | | | | | |
| Jang EJ, Choe YJ, Kim RK, Park YJ. BNT162b2 Vaccine Effectiveness Against the SARS-CoV-2 Omicron Variant in Children Aged 5 to 11 Years. JAMA pediatrics. 2023 Jan 9. | 3,062,281 | Healthy children | This study was part of the Korea COVID-19 Vaccine Effectiveness Study (K-COVE), which was initiated and managed by the Korea Disease Control and Prevention Agency | N/A | <https://jamanetwork.com/journals/jamapediatrics/fullarticle/2800322> | Living Review: McMaster Vaccine Effectiveness Review (<https://www.mcmasterforum.org/docs/default-source/product-documents/living-evidence-syntheses/covid-19-living-evidence-synthesis-8.20---what-is-the-effectiveness-of-available-covid-19-vaccines-for-children-and-adolescents-including-variants-of-concern.pdf?sfvrsn=4e2f4bb6_5>)  14 February 2023 |
| Khan FL, Nguyen JL, Singh TG, Puzniak LA, Wiemken TL, Schrecker JP, Taitel MS, Zamparo JM, Jodar L, McLaughlin JM. Estimated BNT162b2 Vaccine Effectiveness Against Infection With Delta and Omicron Variants Among US Children 5 to 11 Years of Age. JAMA Network Open. 2022 Dec 1;5(12):e2246915-. | 170,803 | Healthy children | Pfizer Inc. | N/A | <https://jamanetwork.com/journals/jamanetworkopen/article-abstract/2799549> | Living Review: McMaster Vaccine Effectiveness Review (<https://www.mcmasterforum.org/docs/default-source/product-documents/living-evidence-syntheses/covid-19-living-evidence-synthesis-8.20---what-is-the-effectiveness-of-available-covid-19-vaccines-for-children-and-adolescents-including-variants-of-concern.pdf?sfvrsn=4e2f4bb6_5>)  14 February 2023 |
| Tartof SY, Frankland TB, Puzniak L, Slezak JM, Hong V, Takhar H, Ogun OA, Simmons S, Xie F, Zamparo J, Ackerson BK. BNT162b2 against COVID-19-associated Emergency Department and Urgent Care Visits among Children 5–11 Years of Age: a Test Negative Design. Journal of the Pediatric Infectious Diseases Society. 2023 Jan 28:piad005. | 3,984 | Healthy children | Pfizer Inc. | N/A | <https://doi.org/10.1093/jpids/piad005> | <https://view-hub.org/covid-19/effectiveness-studies>  14 February 2023 |
