# Supplementary material for: Effectiveness of the BNT162b2 vaccine in preventing morbidity and mortality associated with COVID-19 in children aged 5 to 11 years: A systematic review and meta-analysis
Source: PLOS Glob Public Health. 2023 Dec 4;3(12):e0002676. doi: 10.1371/journal.pgph.0002676 (PMC10695397; doi:10.1371/journal.pgph.0002676)
Supplement: S4 Table — (DOCX) [file pgph.0002676.s005.docx]

**S4 TABLE. CHARACTERISTICS OF EXCLUDED STUDIES**

| **STUDY** | **REASON FOR EXCLUSION** |
| --- | --- |
| Chadeau-Hyam M, Wang H, Eales O, Haw D, Bodinier B, Whitaker M, Walters CE, Ainslie KE, Atchison C, Fronterre C, Diggle PJ. REACT-1 study round 14: High and increasing prevalence of SARS-CoV-2 infection among school-aged children during September 2021 and vaccine effectiveness against infection in England. medRxiv. 2021 Jan 1. | Wrong population |
| Chadeau-Hyam M, Wang H, Eales O, Haw D, Bodinier B, Whitaker M, Walters CE, Ainslie KE, Atchison C, Fronterre C, Diggle PJ. SARS-CoV-2 infection and vaccine effectiveness in England (REACT-1): a series of cross-sectional random community surveys. The Lancet Respiratory Medicine. 2022 Apr 1; 10(4):355-66. | Wrong population |
| Gonzalez SE, Olszevicki S, Gaiano A, Baino AN, Regairaz L, Salazar M, Pesci S, Marin L, Gonzalez V, Varela T, Ceriani L. Effectiveness of BBIBP-CorV, BNT162b2 and mRNA-1273 vaccines against hospitalisations among children and adolescents during the Omicron outbreak in Argentina. medRxiv. 2022 Jan 1. | Wrong intervention |
| Kildegaard H, Lund LC, Højlund M, Stensballe LG, Pottegård A. Risk of adverse events after covid-19 in Danish children and adolescents and effectiveness of BNT162b2 in adolescents: cohort study. BMJ. 2022 Apr 11; 377. | Wrong population |
| Anderegg NT, Althaus C, Colin S, Hauser AW, Laube A, Mäusezahl M, Wagner M, Zaffora B, Riou JY. Assessing real-world vaccine effectiveness against severe forms of SARS-CoV-2 infection: an observational study from routine surveillance data in Switzerland. Swiss medical weekly. 2022 Apr 11;152. | Wrong population |
| Araujo da Silva AR, de Carvalho BR, Esteves MD, Teixeira CH, Souza CV. The role of COVID-19 vaccinal status in admitted children during Omicron variant circulation in Rio de Janeiro, City—Preliminary Report. Vaccines. 2022 Apr 15;10(4):619. | Wrong population |
| Farhat M, Al-Ibrahim R, Almohammedali A, Aljishi R, Alalwan B. Study of the side effects of Pfizer and Oxford COVID-19 vaccines in the eastern province of Saudi Arabia. International Journal of General Medicine. 2022 Jan 1:7547-58. | Wrong population |
| González S, Olszevicki S, Gaiano A, Baino AN, Regairaz L, Salazar M, Pesci S, Marín L, Martínez VV, Varela T, Ceriani L. Effectiveness of BBIBP-CorV, BNT162b2 and mRNA-1273 vaccines against hospitalisations among children and adolescents during the Omicron outbreak in Argentina: A retrospective cohort study. The Lancet Regional Health-Americas. 2022 Sep 1;13:100316. | Wrong intervention |
| Gram MA, Emborg HD, Schelde AB, Friis NU, Nielsen KF, Moustsen-Helms IR, Legarth R, Lam JU, Chaine M, Malik AZ, Rasmussen M. Vaccine effectiveness against SARS-CoV-2 infection or COVID-19 hospitalization with the Alpha, Delta, or Omicron SARS-CoV-2 variant: A nationwide Danish cohort study. PLoS medicine. 2022 Sep 1;19(9): e1003992. | Wrong population |
| Hause AM. Safety monitoring of Pfizer-BioNTech COVID-19 vaccine booster doses among children aged 5–11 years—United States, May 17–July 31, 2022. MMWR. Morbidity and Mortality Weekly Report. 2022;71. | Wrong intervention |
| Lin DY, Gu Y, Wheeler B, Young H, Holloway S, Sunny SK, Moore Z, Zeng D. Effectiveness of Covid-19 vaccines over a 9-month period in North Carolina. New England Journal of Medicine. 2022 Mar 10;386(10):933-41. | Wrong population |
| Mohsin M, Mahmud S, Mian AU, Hasan P, Muyeed A, Ali MT, Ahmed FF, Islam A, Rahman MM, Islam M, Khan MH. Side effects of COVID-19 vaccines and perceptions about COVID-19 and its vaccines in Bangladesh: A cross-sectional study. Vaccine: X. 2022 Dec 1; 12:100207. | Wrong population |
| Molteni E, Canas LS, Kläser K, Deng J, Bhopal SS, Hughes RC, Chen L, Murray B, Kerfoot E, Antonelli M, Sudre CH. Post-vaccination infection rates and modification of COVID-19 symptoms in vaccinated UK school-aged children and adolescents: A prospective longitudinal cohort study. The Lancet Regional Health-Europe. 2022 Aug 1; 19:100429. | Wrong population |
| Rosa Duque JS, Leung D, Yip KM, Lee DH, So HK, Wong WH, Lau YL. Effectiveness of BNT162b2 and CoronaVac against paediatric COVID-19-associated hospitalization and moderate-to-severe disease. medRxiv. 2022:2022-09. | Wrong intervention |
| Sinclair JE, Mayfield HJ, Short KR, Brown SJ, Puranik R, Mengersen K, Litt JC, Lau CL. A Bayesian network analysis quantifying risks versus benefits of the Pfizer COVID-19 vaccine in Australia. NPJ Vaccines. 2022 Aug 11;7(1):93. | Wrong population |
| Sutardi A, Ramatillah DL. Evaluation comparison between sinovac and pfizer vaccine among indonesian children and teenager under 18 years old. Int J App Pharm [Internet]. 2022 Apr. 1 [cited 2023 Feb. 15];14(2):22-30. | Wrong population |
| Tsang NN, So HC, Cowling BJ, Leung GM, Ip DK. Effectiveness of BNT162b2 and CoronaVac COVID-19 vaccination against asymptomatic and symptomatic infection of SARS-CoV-2 omicron BA. 2 in Hong Kong: a prospective cohort study. The Lancet Infectious Diseases. 2022 Dec 12. | Wrong population |
