# Supplementary figures and images for: Effectiveness of the BNT162b2 vaccine in preventing morbidity and mortality associated with COVID-19 in children aged 5 to 11 years: A systematic review and meta-analysis
Source: PLOS Glob Public Health. 2023 Dec 4;3(12):e0002676. doi: 10.1371/journal.pgph.0002676 (PMC10695397; doi:10.1371/journal.pgph.0002676)

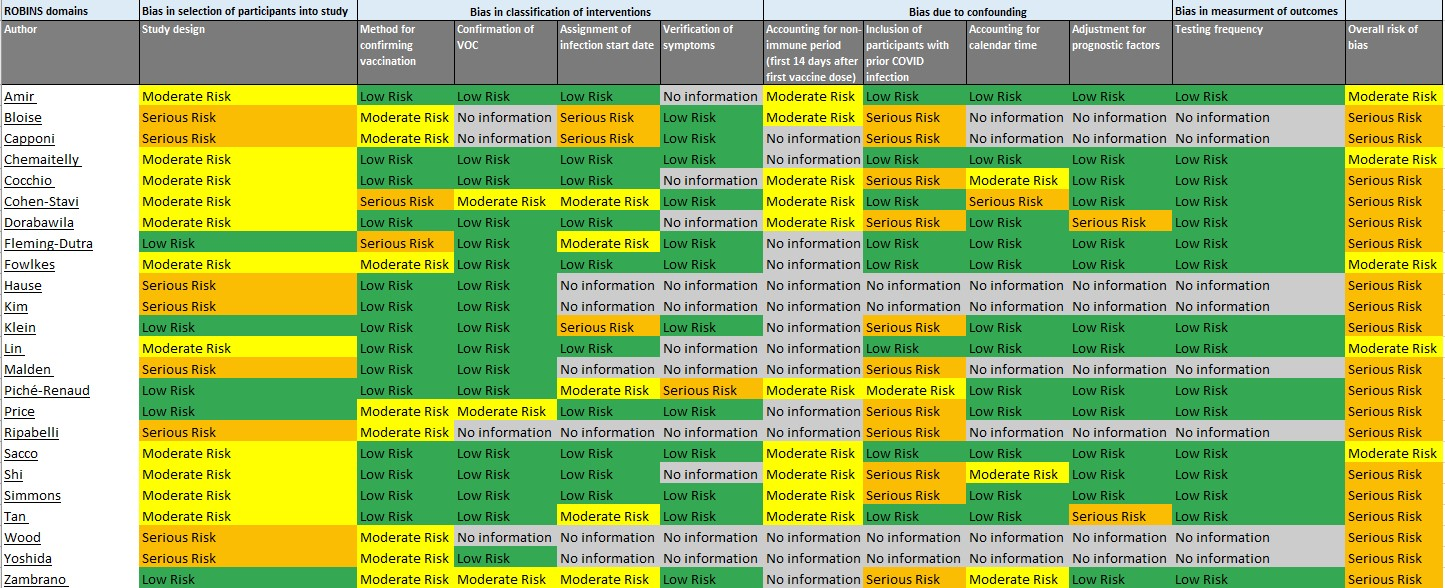

Supplement: S1 Fig — (TIF) [file pgph.0002676.s006.tif]
